# Supplementary material for: Assessment of the lifetime prevalence and incidence of induced abortion and correlates among female sex workers in Mombasa, Kenya: a secondary cohort analysis
Source: BMJ Open. 2022 Oct 7;12(10):e053218. doi: 10.1136/bmjopen-2021-053218 (PMC9557798; doi:10.1136/bmjopen-2021-053218)
Supplement: Author's manuscript [file bmjopen-2021-053218.draft_revisions.pdf]

BMJ Open

BMJ Open is committed to open peer review. As part of this commitment we make the peer review history of every article we publish publicly available.

When an article is published we post the peer reviewers' comments and the authors' responses online. We also post the versions of the paper that were used during peer review. These are the versions that the peer review comments apply to.

The versions of the paper that follow are the versions that were submitted during the peer review process. They are not the versions of record or the final published versions. They should not be cited or distributed as the published version of this manuscript.

BMJ Open is an open access journal and the full, final, typeset and author-corrected version of record of the manuscript is available on our site with no access controls, subscription charges or pay-per-view fees (http://bmjopen.bmj.com).

If you have any questions on BMJ Open's open peer review process please email info.bmjopen@bmj.com

BMJ Open

LIFETIME PREVALENCE AND INCIDENCE OF INDUCED ABORTION AND CORRELATES IN A CLUSTER-RANDOM SAMPLE OF FEMALE SEX WORKERS IN MOMBASA, KENYA

Journal:	*BMJ Open*
Manuscript ID	bmjopen-2021-053218
Article Type:	Original research
Date Submitted by the Author:	10-May-2021

Complete List of Authors:	Simmelink, Anne Marieke; The Aga Khan University - Kenya, Population Health<br>Gichuki, Caroline M.; The Aga Khan University Hospital Nairobi, Department of Population Health; International Centre for Reproductive Health Kenya<br>Ampt, Frances H.; Burnet Institute, Department of Epidemiology and Preventive Medicine; Monash University, Department of Epidemiology and Preventive Medicine<br>Manguro, Griffins; International Centre for Reproductive Health Kenya<br>Lim, Megan; Burnet Institute; Monash University, Department of Epidemiology and Preventive Medicine<br>Agius, Paul; Burnet Institute, Department of Epidemiology and Preventive Medicine; Monash University, Department of Epidemiology and Preventive Medicine<br>Hellard, Margaret; Burnet Institute, Department of Epidemiology and Preventive Medicine; Monash University, Department of Epidemiology and Preventive Medicine<br>Jaoko, Walter; University of Nairobi, Department of Medical Microbiology<br>Stoové, Mark; Burnet Institute, Department of Epidemiology and Preventive Medicine; Monash University, Department of Epidemiology and Preventive Medicine<br>L'Engle, Kelly; University of San Francisco<br>Temmerman, Marleen; International Centre for Reproductive Health Kenya; The Aga Khan University Hospital Nairobi, Department of Obstetrics and Gynaecology<br>Gichangi, Peter; International Centre for Reproductive Health Kenya; Ghent University, Department of Public Health and Primary Care<br>Luchters, Stanley; The Aga Khan University Hospital Nairobi, Department of Population Health; Burnet Institute, Department of Population Health

Keywords:	SEXUAL MEDICINE, PUBLIC HEALTH, EPIDEMIOLOGY

SCHOLARONE™
Manuscripts

I, the Submitting Author has the right to grant and does grant on behalf of all authors of the Work (as defined in the below author licence), an exclusive licence and/or a non-exclusive licence for contributions from authors who are: i) UK Crown employees; ii) where BMJ has agreed a CC-BY licence shall apply, and/or iii) in accordance with the terms applicable for US Federal Government officers or employees acting as part of their official duties; on a worldwide, perpetual, irrevocable, royalty-free basis to BMJ Publishing Group Ltd ("BMJ") its licensees and where the relevant Journal is co-owned by BMJ to the co-owners of the Journal, to publish the Work in this journal and any other BMJ products and to exploit all rights, as set out in our licence.

The Submitting Author accepts and understands that any supply made under these terms is made by BMJ to the Submitting Author unless you are acting as an employee on behalf of your employer or a postgraduate student of an affiliated institution which is paying any applicable article publishing charge ("APC") for Open Access articles. Where the Submitting Author wishes to make the Work available on an Open Access basis (and intends to pay the relevant APC), the terms of reuse of such Open Access shall be governed by a Creative Commons licence – details of these licences and which Creative Commons licence will apply to this Work are set out in our licence referred to above.

Other than as permitted in any relevant BMJ Author's Self Archiving Policies, I confirm this Work has not been accepted for publication elsewhere, is not being considered for publication elsewhere and does not duplicate material already published. I confirm all authors consent to publication of this Work and authorise the granting of this licence.

For peer review only

LIFETIME PREVALENCE AND INCIDENCE OF INDUCED ABORTION AND CORRELATES IN A CLUSTER-RANDOM SAMPLE OF FEMALE SEX WORKERS IN MOMBASA, KENYA

A. Marieke Simmelink[1], Caroline M. Gichuki[1,2], Frances H. Ampt[3,4], Griffins Manguro[2], Megan S.C. Lim[3,4], Paul A. Agius[3,4], Margaret Hellard[3,4,5,6], Walter Jaoko[7], Mark Stoové[3,4,8], Kelly L'Engle[9], Marleen Temmerman[2,10,11], Peter Gichangi[2,10,12], Stanley Luchters[1,3,4,10]

AFFILIATIONS

1. Department of Population Health, Medical College, Aga Khan University, Nairobi, Kenya
2. International Centre for Reproductive Health (ICRH), Mombasa, Kenya
3. Burnet Institute, Melbourne, Australia
4. Department of Epidemiology and Preventive Medicine, Monash University, Melbourne, Australia
5. Department of Infectious Diseases, The Alfred Hospital, Melbourne, Australia
6. Doherty Institute and School of Population and Global Health, University of Melbourne, Australia
7. Department of Medical Microbiology, University of Nairobi, Nairobi, Kenya
8. School of Psychology and Public Health, La Trobe University, Melbourne, Australia
9. University of San Francisco, San Francisco, California, USA
10. Department of Public Health and Primary Care, Ghent University, Ghent, Belgium
11. Department of Obstetrics and Gynaecology, Aga Khan University, Kenya
12. Technical University of Mombasa, Mombasa, Kenya

CORRESPONDING AUTHOR

Stanley Luchters, Chair of the Department of Population Health, Medical College, Aga Khan University, Nairobi, Kenya. Email: stanley.luchters@aku.edu

Word count: 2740

For peer review only

STRUCTURED ABSTRACT

Introduction
Prevalence of lifetime induced abortion in female sex workers (FSWs) in Kenya was previously estimated between 43 and 86%. Our secondary objective aimed to assess lifetime prevalence and correlates, and incidence and predictors of induced abortions among FSWs in Kenya.

Methods
Data was collected as part of the WHISPER or SHOUT cluster-randomized trial in Mombasa, assessing effectiveness of an SMS-intervention to reduce incidence of unintended pregnancy. Eligible participants were current FSWs, 16-34 years and not pregnant or planning pregnancy. Baseline data on self-reported lifetime abortion, correlates and predictors were collected between September 2016 and May 2017. Abortion incidence was measured at six- and twelve-months follow-up. A multivariable logistic regression model was used to assess correlates of lifetime abortion and discrete-time survival analysis was used to assess predictors of abortions during follow-up.

Results
Among 866 eligible participants with available data on outcome and exposure variables, lifetime abortion prevalence was 11.9%, while lifetime unintended pregnancy prevalence was 51.2%. Correlates of lifetime abortions were currently not using a highly effective contraceptive (AOR=1.76 [95%CI=1.11-2.79] p=0.017) and having ever experienced intimate partner violence (IPV) (AOR=2.61 [95%CI=1.35-5.06] p=0.005). Incidence of unintended pregnancy and induced abortion were 15.5 and 3.9 per 100 women-years, respectively. No statistically significant associations were found between hazard of abortion and age, sex work duration, partner status, contraceptive use and IPV experience.

Conclusion
Although experience of unintended pregnancy remains high, lifetime prevalence of abortion may have decreased among FSW in Kenya. Addressing IPV could further decrease induced abortions in this population.

KEYWORDS
Sex Work, induced abortion, unintended pregnancy, contraception, cluster-random sample, Kenya

FUNDING SUPPORT
Australia's National Health and Medical Research Council (NHMRC).

For peer review only

ARTICLE SUMMARY

Strengths and limitations

- This study presents incidence of abortion in a cluster-randomised cohort of FSWs.
- It is the first to analyse predictors of abortions in FSWs, rather than correlates of past abortions only.
- This paper explores a secondary research question, and the study was not originally powered to assess the predictors of abortions during follow-up.
- The sensitive topic of abortions and SRH in general, might have resulted in a social desirability bias.

For peer review only

For peer review only - http://bmjopen.bmj.com/site/about/guidelines.xhtml

INTRODUCTION

Research findings show that about 5% of Kenya's urban female reproductive population could be involved in sex work.[1] Female sex workers (FSWs) experience higher than average rates of HIV, other sexually transmitted infections (STIs) and unintended pregnancy.[2–5]

Unintended pregnancies often have negative consequences for FSWs, including financial adversity, social stigma and induced abortion.[4] In countries where abortion is illegal or difficult to access, women frequently resort to unsafe practices, risking severe medical complications.[6]

Reported prevalence of lifetime abortion among FSWs in low- and middle-income countries (LMICs) varies from 24% in Laos in 2012 to 86% in 2000/01 in Central and Western Kenya.[7–16] Many of these abortions are unsafe or sought in the informal sector.[8,11,16] In Mombasa, lifetime abortion among FSWs was estimated at 43% in 2008.[17]

In 2010, Kenya liberalized its abortion law, making abortion legal when "there is need for emergency treatment, or the life or health of the mother is in danger".[18] In practice, older laws criminalizing abortion remain in place, creating ambiguity among health professionals with lawsuits remaining a threat.[19] Moreover, social, cultural and religious beliefs condemning pregnancy termination, misconceptions about the illegality and costs of the procedure, still hamper access to safe services for women.[4,19]

Previous articles recognized the need for longitudinal data about abortions in FSWs.[8,14] Identifying predictors for induced abortions will help inform future policies to improve care around abortions for FSWs.

This secondary data analysis aimed to examine lifetime prevalence and correlates, and incidence and predictors of induced abortions in a cluster-random sample of FSWs in Mombasa, Kenya.

For peer review only - http://bmjopen.bmj.com/site/about/guidelines.xhtml

MATERIAL AND METHODS

Study design

This study analysed data collected in the WHISPER or SHOUT trial. A detailed description of the study protocol can be found elsewhere.[20] In summary, the study was a two-arm cluster-randomized controlled trial assessing the effectiveness of two SMS-based interventions targeting sexual and reproductive health (SRH) and nutrition in FSWs in Mombasa, Kenya. The primary aim of the SRH intervention was to reduce the incidence of unintended pregnancy among FSWs.

The study was conducted in two sub-counties in Mombasa, Kisauni and Changamwe, between September 2016 and July 2018. Ninety-three venues were randomly sampled with a probability proportional to FSW population size at the venue (Figure 1). Trained community mobilisers and peer educators recruited FSWs from the venues until the required sample size of 860 FSWs was achieved. Sex-work venues were randomized to either the SRH or nutrition intervention group after the cluster was fully enrolled and baseline data obtained.

The study was approved by the Kenyatta National Hospital and University of Nairobi Ethics and Research Committee, Kenya (KNH-UoN ERC—KNH-ERC/RR/493) and the Monash University Human Research Ethics Committee, Australia (MUHREC—CF16/1552—2016000812).

Study participants and procedures

Women were eligible for the study if they were aged 16-34 years; self-reported to have engaged in paid sex work in the last 6 months; were not pregnant or planning pregnancy within the next 12 months; resided within the study area; and were able to read text messages in basic English. Study-specific community mobilizers visited the selected clusters to recruit FSWs and conducted pre-screening interviews. Eligible FSWs were referred to the nearest study clinic for a clinical assessment, including a urine pregnancy test, and STI and HIV testing. Enrolled participants then completed a structured questionnaire administered in Swahili by trained research assistants, who had previously participated in research with FSWs. The questionnaire captured detailed sociodemographic, sexual and reproductive health information. Follow-up visits were scheduled at 6 and 12 months after enrolment. Procedures at follow-up visits were similar to those done at enrolment.

Participants received two to three SMS messages per week for 12 months. The messages consisted of stand-alone push messages, role model stories and on-demand messages, accessed using assigned codes. Participants only received and accessed messages on their phones from their assigned intervention.

Outcomes

For peer review only

Lifetime prevalence of induced abortion was assessed at baseline with the question 'How many times have you ever had an induced abortion?'. Induced abortions were assessed during follow-up, by asking participants if they had been pregnant since their last visit. The outcome of each reported pregnancy was then assessed, and in the case of an induced abortion, the location was documented. Formal sector abortions were those taking place at a government or private hospital/clinic, a private doctor/GP or an FP clinic. Informal sector abortions were defined as those taking place at home, a pharmacy or traditional healer.[21] Pregnancies during follow-up were confirmed with a urine pregnancy test at the study clinic, or self-reported by the participant when occurring between study visits. Pregnancy intention for all reported pregnancies was assessed using the London Measure of Unintended Pregnancy (LMUP), a six-item scale. A pregnancy scoring less than 10 out of 12 on the LMUP was defined as unintended.[22]

All correlates of lifetime induced abortions and predictors of incident induced abortions were self-reported at baseline. Use of a highly effective contraceptive method was defined as use of contraceptive implants, IUD, injection, oral contraceptive pill and sterilization. High knowledge on FP was defined as answering five out of six true-false statements on FP correctly. Having a positive attitude on FP was defined as agreeing with at least three out of four positive attitude statements. Self-efficacy in FP was defined as high when agreeing with two statements on this topic. Household socio-economic status tertiles were generated using principal component analysis, based on 12 household assets. Prevalence of intimate partner violence (IPV) was assessed by asking if the participant had ever been pushed, slapped, hit or kicked by a partner, or had been physically forced to have sex, agreed to sex out of fear of the consequences or forced to do something sexual that she perceived as degrading or humiliating. For each item, it was also assessed if this happened in the previous 12 months.

Statistical analysis

Statistical analysis was performed using Stata software version 14 (StataCorp, College Station, TX, USA). Covariates for the multivariate models were determined on the basis of a review of literature or theoretical assumptions by the co-authors. Correlates of lifetime abortion were identified using weighted multivariable logistic regression. Associations were considered statistically significant at the 5% level. The outcome incident abortion was interval-censored (measured at 6-monthly intervals). Therefore, a discrete-time survival analysis was performed using generalized linear mixed (GLM) modelling with complementary log-log link function and binomial distribution, a method that produces estimated hazard ratios. Abortions during follow-up were analysed for all participants who attended at least one follow-up visit. All outcomes presented here are cluster-adjusted, based on inverse probability sample means to account for sampling bias.

For peer review only

Patient and public involvement

Patients or the public were not directly involved in the design, or conduct, or reporting, or dissemination plans of our research.

For peer review only

RESULTS

During the recruitment period of 14 September 2016 to 16 May 2017, 882 women were enrolled in the study. Follow-up continued until 31 July 2018. A sub-sample of 866 women (98.2%) was analysed for this paper (Figure 1).

Mean age was 25.5 years (SD=4.7) (Table 1). The majority of women (n=765, 88.7%) had at least completed primary education and 306 (34.9%) had completed secondary education. Just over half of the participants (n=484, 56.6%) reported to have a current husband or boyfriend, but 812 (94.0%) reported not to live with a partner. Mean duration of employment in sex work was 4.7 years (SD=3.5). Among women currently reporting a husband or boyfriend, 344/483 (70.8%) had not disclosed their employment in sex work to their partners. 605/861 women (68.9%) worked fulltime in sex work and 508/863 (59.8%) earned more than 2000 Ksh (about USD $20) per week from sex work. The majority of women (n=666, 76.1%) had ever been pregnant, and 451 (51.2%) ever had an unintended pregnancy. 103/866 (11.9%) reported to have had at least one induced abortion in their lifetime. Among women who had an induced abortion, 58/102 (57.1%) went to a private hospital or clinic for the most recent abortion. 29/102 (29.1%) women had their most recent abortion in the informal sector, like home, a pharmacy or traditional healer. At baseline, 463 women (54.4%) reported to use a highly effective contraceptive method. Three-quarters of FSW (650/866; 75.0%) ever experienced IPV and 525/866 (60.1%) experienced IPV in the past 12 months the before the baseline questionnaire was conducted.

Table 1. Socio-demographic characteristics, reproductive history, contraceptive use and intimate partner violence at baseline of female sex workers in Mombasa, Kenya (n=866, unless stated otherwise)

Characteristic	n	Cluster-adjusted mean (SD) or proportion in % (95% CI)[a]
Mean age, in years		25.5 (4.7)
Highest level of education		
None or some primary	101	11.2 (9.2-13.6)
Completed primary or some secondary	459	53.8 (50.1-57.6)
Completed secondary or some tertiary	306	34.9 (31.3-38.7)
Religion (n=864)		
Protestant	389	44.8 (41.4-48.2)
Catholic	304	36.0 (32.3-39.8)
Muslim	171	19.2 (15.6-23.4)
Electricity available in household (n=863)[b]	660	76.4 (73.0-79.5)
Duration of sex work, in years		4.7 (3.5)

For peer review only - http://bmjopen.bmj.com/site/about/guidelines.xhtml

Fulltime FSW (n=861)[c]	605	68.9 (64.0-73.5)
Weekly income from sex work (n=861)		
≤1000 Ksh	144	16.2 (13.2-19.8)
1001-2000 Ksh	211	24.0 (21.0-27.3)
≥2001 Ksh[d]	508	59.8 (55.1-64.3)
Sex work venue		
Bar with lodging	388	43.8 (37.7-50.2)
Bar without lodging	147	17.2 (13.7-21.3)
Lodging/guesthouse	138	15.1 (10.9-20.5)
Street/beach	86	11.2 (7.8-15.7)
Other[e]	107	12.7 (9.1-17.5)
Marital status		
Married/cohabiting	54	6.0 (4.5-8.0)
Single (not cohabiting)	627	73.0 (69.6-76.2)
Separated/divorced/widowed	185	21.0 (18.0-24.3)
Currently has husband/boyfriend	484	56.6 (52.5-60.7)
Disclosure of sex work to husband/boyfriend (n=483)[f]		
Yes	136	28.7 (24.4-33.3)
No	344	70.8 (66.0-75.1)
Don't know	3	0.6 (0.2-1.7)
Ever had a pregnancy	666	76.1 (72.3-79.5)
Has a living child	622	71.2 (67.1-75.0)
Ever had an unintended pregnancy (N=864)[g]	451	51.2 (47.4-54.9)
Ever had an induced abortion	103	11.9 (10.0-14.2)
Location of most recent induced abortion (N=102)		
Government hospital	2	1.8 (0.4-6.9)
FP clinic, like Marie Stopes	9	8.3 (4.4-15.0)
Private hospital/clinic	58	57.1 (46.5-67.2)
Private doctor GP	4	3.7 (1.4-9.6)
Pharmacy	9	8.9 (4.6-16.8)
Traditional healer	4	3.8 (1.4-10.0)
Home	16	16.4 (9.0-27.8)
Uses a highly effective contraceptive method[h]	473	54.4 (49.5-59.2)
Ever experienced IPV[i]	650	75.0 (71.1-78.5)
Experienced IPV in past 12 months[j]	525	60.1 (55.5-64.6)

[a] Inverse probability-weighted percentages. [b] Availability of electricity in the household is presented here as a proxy for household SES. [c] Fulltime work as FSW is characterized as having no other sources of income in the last 6 months. [d] 1000 Kenyan Shilling (Ksh) is about USD $10. [e] Brothel, strip club, casino, massage parlors, parks or home. [f] Among participants with a husband or boyfriend. [g] Assessed using the London Measure of Unintended Pregnancy (LMUP) [h] Highly effective is defined as use of contraceptive implants, IUD, injection, oral contraceptive pill and sterilization. [i] IPV= intimate partner violence. [j] Before baseline.

Women currently not using a highly effective contraceptive (AOR=1.76 [95%CI=1.11-2.79] p=0.017) and women who ever experienced IPV (AOR=2.61 [95%CI=1.35-5.06] p=0.005) were significantly more likely to report a history of induced abortion, when controlled for

For peer review only - http://bmjopen.bmj.com/site/about/guidelines.xhtml

potential confounders (Table 2). Longer duration of sex work showed a borderline positive association with history of abortion (AOR=1.08 [95%CI=1.00-1.16] p=0.053). Although higher age was significantly associated with a history of abortion in bivariate analysis, after adjusting for confounding factors this association was no longer seen.

Table 2. Correlates of participants with a history of induced abortion, and cluster-adjusted bi- and multivariable logistic regression analysis on history of induced abortion (n=866)

Characteristic		Crude Odds Ratio (OR)		Adjusted OR	
	**Ever had an induced abortion (n=103); n/N (cluster-adjusted proportion in %)[a]**	**OR (95% CI)[b]**	**p-value**	**OR (95% CI)[b]**	**p-value**
Age (in years)	27.0 (4.9)[c]	1.08 (1.03-1.14)	**0.001**	1.04 (0.97-1.11)	0.280
Highest level of education					
None or some primary	14/101 (13.5)	Ref.		Ref.	
Completed primary or some secondary	57/459 (12.4)	0.90 (0.47-1.72)	0.749	0.96 (0.48-1.99)	0.895
Completed secondary or some tertiary	32/306 (10.7)	0.77 (0.40-1.47)	0.423	0.83 (0.40-1.84)	0.620
SES-tertile[e]					
Poorest	39/290 (13.4)	Ref.		Ref.	
Middle	33/287 (10.9)	0.79 (0.46-1.35)	0.386	0.89 (0.50-1.59)	0.697
Richest	31/289 (11.4)	0.83 (0.47-1.46)	0.510	0.83 (0.46-1.50)	0.674
Mean duration of sex work (in years)	6.1 (3.4)[c]	1.12 (1.07-1.17)	**<0.001**	1.08 (1.00-1.16)	0.053
Highly effective contraceptive use[d]					
Yes	51/473 (10.6)	Ref.		Ref.	
No	52/393 (13.5)	1.32 (0.88-1.97)	0.173	1.76 (1.11-2.79)	**0.017**
High FP knowledge score					
No	60/562 (10.5)	Ref.		Ref.	
Yes	43/304 (14.5)	1.44 (0.95-2.19)	0.084	1.34 (0.85-2.10)	0.200
Positive attitude to FP use					
No	43/354 (12.4)	Ref.		Ref.	
Yes	60/512 (11.6)	0.93 (0.60-1.44)	0.743	0.90 (0.56-1.45)	0.661
High FP-specific self-efficacy					
No	24/237 (10.1)	Ref.		Ref.	
Yes	79/629 (12.6)	1.28 (0.76-2.17)	0.345	1.23 (0.72-2.10)	0.454
Ever experienced intimate partner violence					
No	12/216 (5.2)	Ref.		Ref.	
Yes	91/650 (14.1)	2.98 (1.55-5.74)	**0.001**	2.61 (1.35-5.06)	**0.005**

[a] Inverse probability-weighted percentages. [b] Standard errors are corrected by cluster sandwich variance estimation. [c] Mean (SD) of women who ever had an induced abortion. [d] Highly effective is defined as use of contraceptive implants, IUD, injection, oral contraceptive pill and sterilization. [e] SES = Socio-economic status.

During the study follow-up, 773 women attended at least one follow-up visit (Figure 1). A total of 131 participants became pregnant, with a total of 145 pregnancies among these women (Figure 2). Of these pregnancies, 122/145 were unintended according to the LMUP.

For peer review only

Among 145 pregnancies, 31 ended in induced abortion, among 29 women and across 789 women-years at risk. Overall incidence rate was 3.9 induced abortions per 100 women-years of observation. Out of 31 abortions, 19 took place in the formal sector and 12 in an informal setting.

The GLM modelling of abortion incidence showed that women experiencing IPV in the past year (HR=1.93 [95%CI=0.86-4.34] p=0.122) and women not using a highly effective contraceptive (HR=1.51 [95%CI=0.66-3.49] p=0.332) exhibited a higher hazard of abortion, independent of other factors, although these results were not significant (Table 3). We did not find a relation between age, mean duration of sex work, currently having a husband or boyfriend and the intervention under study and hazard of induced abortion.

Table 3. Baseline predictors of incident abortion in FSWs among Mombasa, Kenya (N=773)[a]

**Baseline predictors of incident abortions**	**Unadjusted[b]**		**Adjusted HR[c]**	
	**HR (95% CI)[d]**	**p-value**	**HR (95% CI)[d]**	**p-value**
Age (in years)	0.96 (0.89-1.04)	0.315	1.00 (0.91-1.09)	0.918
Mean duration of sex work (in years)	0.93 (0.82-1.05)	0.234	0.92 (0.79-1.09)	0.336
Currently has husband/boyfriend	0.83 (0.40-1.73)	0.622	0.80 (0.39-1.64)	0.537
Not using highly effective contraceptive[e]	1.50 (0.69-3.23)	0.310	1.51 (0.66-3.49)	0.332
Experienced IPV in last 12 months	1.67 (0.74-3.79)	0.216	1.93 (0.86-4.34)	0.122

[a] Discrete-time survival analysis including the first induced abortion per women. Generalized linear mixed model with complementary log-log link, binomial distribution, offset for log time between visits and random intercept for participants. [b] HR = Hazard Ratio. [c] All adjusted Hazard Ratios are also adjusted for the intervention. The intervention had no detectable effect on the outcome of incident abortions. [d] Cluster robust standard errors for sex-work venue clustering. [e] Highly effective is defined as use of contraceptive implants, IUD, injection, oral contraceptive pill and sterilization.

For peer review only

DISCUSSION

This study adds to the current knowledge of abortion practices in FSWs. Lifetime induced abortion prevalence in this population was 11.9%. This seems considerably lower than previous figures of lifetime abortion of 86% in 2004 in central and western Kenya among FSWs of a similar age, and 43% in 2008 in Mombasa among FSWs who were on average 2 years older.[7,17] In the former study, it was not specified if these abortions also included spontaneous abortions, which might have overestimated the prevalence of abortions. However, despite the sociodemographic and methodological differences between the studies, the size of the difference is suggestive of an actual reduction in abortions in this population. The prevalence of abortions found here is also lower than reports from other LMICs ranging between 24% and 64% in Laos and Cote d'Ivoire, respectively.[8–16] A possible explanation for this lower prevalence is the relatively high use of highly effective contraceptives of 54% in our cohort, compared to similar studies from LMICs. [8,12,14–16] The findings are furthermore consistent with a lower-than-expected HIV prevalence and unintended pregnancy incidence in our cohort and could be a result of peer-mediated interventions implemented over the past years in the Mombasa area.[23,24] These have mostly targeted prevention of HIV and STIs, but likely have had a lowering effect on unintended pregnancies and induced abortions as well.[20,23] Furthermore, this study, in contrast to above referenced studies, attempted to draw a representative sample of a FSW population from community settings, whereas other studies used non-probability sampling methods, which might have overestimated past abortions.

Despite the lower-than-expected unintended pregnancy incidence, still 51% of FSWs in our cohort reported an unintended pregnancy in their lifetime. The gap between lifetime unintended pregnancies and lifetime induced abortions could indicate a high unmet need for induced abortion among FSWs, for example due to ongoing or increasing difficulties in accessing SRH or abortion services for this group or increasing sociocultural barriers to abortion.

The present study is one of the few studies to report incidence of abortion among FSWs and to our knowledge, the first to analyse predictors of abortions in FSWs, rather than correlates of past abortions only. Incidence of induced abortion in our cohort was 3.9 per 100 women-years. Compared to other studies from LMICs, this is similar to two studies reporting abortion incidence rates of 3.1 and 3.0 per 100 women-years and lower than a third study reporting 7.4 induced abortions per 100 women-years among FSWs.[25] The intervention under study had no measurable effect on unintended pregnancy incidence and is therefore unlikely to have affected incidence of induced abortions.[24]

Informal sector abortions where common in this cohort, with 30% of women having had their most recent abortion in the informal sector, and 39% of the reported abortions during follow-

For peer review only - http://bmjopen.bmj.com/site/about/guidelines.xhtml

up happening in the informal sector. These informal sector abortions, put women at higher risk of complications due to unsafe practices and this denotes a need for information on safer alternatives, like the Marie Stopes clinics.[21]

Multiple studies have found both age and duration of sex work to be correlated to past abortions. Commonly higher age [8,11,15] and longer duration [9,12] of sex work were associated with higher lifetime abortion prevalence. One study found that younger age was associated with past abortions.[10] In our cohort, although FSWs with a past abortion in our cohort were older in the crude analysis, after adjusting for other correlates, this difference was no longer significant. The association with longer duration of sex work remained borderline significant in multivariate analysis. We did not find a relation between age and mean duration of sex work and having an induced abortion during follow-up. This might suggest that the association between past abortions and higher age and longer duration of sex work is caused by cumulative exposure to high risk of pregnancies and abortion.

We found a positive association between currently not using a highly effective contraceptive and having a past abortion. No difference was found in FP-specific self-efficacy or knowledge, or attitude towards FP among women with and without a past abortion. The found association could indicate significant barriers to uptake or continuation of a highly effective contraceptive method post abortion, as has previously been acknowledged by a study in Kenya.[26]

In our cohort, experience of IPV was high and the odds of having had a past abortion were more than 2.5-times as high for women who experienced IPV in the past, consistent with findings from other studies.[14,15] Our study also showed a positive association between experience of IPV in the past twelve months and abortions during follow-up, but this was not significant. Experience of (intimate partner) violence has been shown to have a negative effect on the reproductive health of FSWs, with greater risks of adverse pregnancy outcomes and forced termination of pregnancy.[27,28] Addressing the problem of IPV in this population could further lower induced abortions.

Limitations

Some limitations should be considered when interpreting the findings from this study. The sensitive topic of abortions and SRH in general, might have resulted in a social desirability bias. To minimize this, peer-educators and research assistants had previous experience working with the target population and received additional training. Attrition bias might have occurred due to loss to follow-up of pregnant participants, as has been recognized by anecdotal evidence.[24] This might have resulted in an underestimation of abortions in our study. A further limitation is that this paper explores a secondary research question, and the study was not originally powered to assess the predictors of abortions during follow-up.

For peer review only - http://bmjopen.bmj.com/site/about/guidelines.xhtml

Unknown timing of the past abortions in relation to studied correlates, restrict judgement of temporality of the studied associations. Lastly, measurements of abortions stopped when the intervention stopped, so the actual number of abortions during follow-up might in fact be higher than captured in the study.

Suggestions for further research

Future research is needed to explore the trend in abortion incidence among FSWs in Kenya. In order to improve care, we need to better understand current abortion practices, the decision-making process around terminating unintended pregnancies, how uptake of highly effective contraceptives can be increased post-abortion, as well as the relationship between experience of IPV and induced abortions.

CONCLUSIONS

In conclusion, the prevalence of lifetime induced abortions in a random cohort of FSWs in Mombasa, was 11.9% and incidence was 3.9 per 100 women-years, whereas prevalence and incidence of unintended pregnancies were higher at 51% and 15.5 per 100 women-years, respectively. A history of induced abortion was positively associated with not using a highly contraceptive method at baseline and having experienced IPV in the past. This was, to our knowledge the first study attempting to identify predictors of abortions in FSWs, however the study did not find a significant association with the studied predictors of abortions.

For peer review only

ACKNOWLEDGEMENTS

The authors would like to acknowledge the hard work of the field research team, specifically Christine Maghanga, Millicent Okello, Betty Kitili, Judith Wamaua, Elizabeth Bilasi, Marion Mwangi, Promillah Muindi, Rukia Abdallah and Khadija Kassim, as well as all the study participants of the study. The authors gratefully acknowledge the contribution of funding from Australia's National Health and Medical Research Council for the WHISPER or SHOUT trial (Project Grant GNT 1087006), Career Development Fellowships for SL, Senior Research Fellowship for MS, and a Postgraduate Scholarship for FHA. The sponsor did not contribute to study design; data collection, analysis, or interpretation; manuscript writing; or the decision to submit the article for publication. Lastly, we acknowledge the contribution of funding from the Victorian Operational Infrastructure Support Program received by the Burnet Institute.

TRIAL REGISTRATION

The trial was registered in the Australian New Zealand Clinical Trials Registry, ACTRN12616000852459.

AUTHOR CONTRIBUTORSHIP

SL was the Principal Investigator on the study. SL, FHA, PG, MSCL, PAA, MH, WJ, MS, KL, MT and MFC contributed to the study design. CG and GM coordinated the trial and undertook data acquisition in Kenya under the supervision of PG. AMS and SL conceptualized the manuscript. AMS and PAA conducted the statistical analyses. AMS and CG wrote the first draft of the manuscript. All authors contributed to data interpretation, provided critical input, and approved the final version of the manuscript.

DECLARATION OF INTERESTS

The authors have no conflicts of interest.

PATIENT AND PUBLIC INVOLVEMENT

Patients or the public were not directly involved in the design, or conduct, or reporting, or dissemination plans of our research.

DATA AVAILABILITY STATEMENT

The original data are not available in a public repository. The corresponding author is to be contacted for the consideration of any data requests.

For peer review only

FIGURE CAPTIONS

Figure 1: Eligibility flow diagram for the WHISPER or SHOUT study as per Consort 2010 statement: extension to cluster randomized trials.[29]

Figure 2: Overview of pregnancy outcomes during the 12-month follow-up. N=773

For peer review only

For peer review only - http://bmjopen.bmj.com/site/about/guidelines.xhtml

REFERENCES

1 Odek WO, Githuka GN, Avery L, *et al.* Estimating the size of the female sex worker population in Kenya to inform HIV prevention programming. *PLoS One* 2014;**9**. doi:10.1371/journal.pone.0089180

2 Baral S, Beyrer C, Muessig K, *et al.* Burden of HIV among female sex workers in low-income and middle-income countries: A systematic review and meta-analysis. *Lancet Infect Dis* 2012;**12**:538–49. doi:10.1016/S1473-3099(12)70066-X

3 Cwikel JG, Lazer T, Press F, *et al.* Sexually transmissible infections among female sex workers: An international review with an emphasis on hard-to-access populations. *Sex Health* 2008;**5**:9–16. doi:10.1071/SH07024

4 Luchters S, Bosire W, Feng A, *et al.* 'A baby was an added burden': Predictors and consequences of unintended pregnancies for female sex workers in Mombasa, Kenya: A mixed-methods study. *PLoS One* 2016;**11**:1–20. doi:10.1371/journal.pone.0162871

5 Ippoliti NB, Nanda G, Wilcher R. Meeting the Reproductive Health Needs of Female Key Populations Affected by HIV in Low- and Middle-Income Countries: A Review of the Evidence. *Stud Fam Plann* 2017;**48**:121–51. doi:10.1111/sifp.12020

6 World Health Organization. WHO | Unsafe abortion: global and regional estimates of the incidence of unsafe abortion and associated mortality in 2008. 2014. doi:10.1017/CBO9781107415324.004

7 Elmore-Meegan M, Conroy RM, Agala CB. Sex Workers in Kenya, Numbers of Clients and Associated Risks: An Exploratory Survey. *Reprod Health Matters* 2004;**12**:50–7. doi:10.1016/S0968-8080(04)23125-1

8 Schwartz S, Papworth E, Thiam-Niangoin M, *et al.* An urgent need for integration of family planning services into HIV care: The high burden of unplanned pregnancy, termination of pregnancy, and limited contraception use among female sex workers in Côte d'Ivoire. *J Acquir Immune Defic Syndr* 2015;**68**:S91–8. doi:10.1097/QAI.0000000000000448

9 Bautista CT, Mejía A, Leal L, *et al.* Prevalence of lifetime abortion and methods of contraception among female sex workers in Bogota, Colombia. *Contraception* 2008;**77**:209–13. doi:10.1016/j.contraception.2007.11.013

10 Chanda MM, Ortblad KF, Mwale M, *et al.* Contraceptive use and unplanned pregnancy among female sex workers in Zambia. *Contraception* 2017;**96**:196–202. doi:10.1016/j.contraception.2017.07.003

11 Lau JTF, Mui LWH, Tsui HY, *et al.* Prevalence of induced abortion and associated factors among Chinese female sex workers in Hong Kong. *J Sex Marital Ther* 2007;**33**:19–29. doi:10.1080/00926230600998441

12 Todd CS, Alibayeva G, Sanchez JL, *et al.* Utilization of contraception and abortion and its relationship to HIV infection among female sex workers in Tashkent, Uzbekistan. *Contraception* 2006;**74**:318–23. doi:10.1016/j.contraception.2006.04.006

13 Zhang XD, Kennedy E, Temmerman M, *et al.* High rates of abortion and low levels of contraceptive use among adolescent female sex workers in Kunming, China: A cross-sectional analysis. *Eur J Contracept Reprod Heal Care* 2014;**19**:368–78. doi:10.3109/13625187.2014.927421

14 Zhang XD, Myers S, Yang HJ, *et al.* Prevalence and correlates of sexual and gender-based violence against Chinese adolescent women who are involved in commercial sex: A cross-sectional study. *BMJ Open* 2016;**6**. doi:10.1136/bmjopen-2016-013409

15 Karamouzian M, Mirzazadeh A, Shokoohi M, *et al.* Lifetime abortion of female sex workers in Iran: Findings of a national bio-behavioural survey In 2010. *PLoS One* 2016;**11**:1–12. doi:10.1371/journal.pone.0166042

16 Cleeve A, Phrasisombath K, Sychareun V, *et al.* Attitudes and experiences regarding induced abortion among female sex workers, Savannakhet Province, Laos. *Sex Reprod Healthc* 2014;**5**:137–41. doi:10.1016/j.srhc.2014.03.001

For peer review only

For peer review only - http://bmjopen.bmj.com/site/about/guidelines.xhtml

17 Sutherland EG, Alaii J, Tsui S, *et al.* Contraceptive needs of female sex workers in Kenya - A cross-sectional study. *Eur J Contracept Reprod Heal Care* 2011;**16**:173–82. doi:10.3109/13625187.2011.564683

18 Constitution of Kenya. Laws of kenya. *Natl Counc Law Report* 2010;:191. doi:10.1364/OE.17.019075\r186571 [pii]

19 Ushie BA, Juma K, Kimemia G, *et al.* Community perception of abortion, women who abort and abortifacients in Kisumu and Nairobi counties, Kenya. *PLoS One* 2019;**14**:1–13. doi:10.1371/journal.pone.0226120

20 Ampt FH, Mudogo C, Gichangi P, *et al.* WHISPER or SHOUT study: Protocol of a cluster-randomised controlled trial assessing mHealth sexual reproductive health and nutrition interventions among female sex workers in Mombasa, Kenya. *BMJ Open* 2017;**7**. doi:10.1136/bmjopen-2017-017388

21 Chemlal S, Russo G. Why do they take the risk? A systematic review of the qualitative literature on informal sector abortions in settings where abortion is legal. *BMC Womens Health* 2019;**19**:1–11. doi:10.1186/s12905-019-0751-0

22 Hall J, Barrett G, Copas A, *et al.* London Measure of Unplanned Pregnancy: guidance for its use as an outcome measure. *Patient Relat Outcome Meas* 2017;**Volume 8**:43–56. doi:10.2147/prom.s122420

23 Manguro GO, Gichuki C, Ampt FH, *et al.* HIV infections among female sex workers in Mombasa, Kenya: current prevalence and trends over 25 years. *Int J STD AIDS* 2020;**31**:1389–97. doi:10.1177/0956462420950571

24 Ampt FH, Lim MSC, Agius PA, *et al.* Effect of a mobile phone intervention for female sex workers on unintended pregnancy in Kenya (WHISPER or SHOUT): a cluster-randomised controlled trial. *Lancet Glob Heal* 2020;**8**:e1534–45. doi:10.1016/S2214-109X(20)30389-2

25 Ampt FH, Willenberg L, Agius PA, *et al.* Incidence of unintended pregnancy among female sex workers in low-income and middle-income countries: a systematic review and meta-analysis. *BMJ Open* 2018;**8**:21779. doi:10.1136/bmjopen-2018-021779

26 Makenzius M, Faxelid E, Gemzell-danielsson K, *et al.* Contraceptive uptake in post abortion care — Secondary outcomes from a randomised controlled trial , Kisumu , Kenya. 2018;**354**:1–13.

27 Swain SN, Saggurti N, Battala M, *et al.* Experience of violence and adverse reproductive health outcomes, HIV risks among mobile female sex workers in India. *BMC Public Health* 2011;**11**:357. doi:10.1186/1471-2458-11-357

28 McDougal L, Strathdee SA, Rangel G, *et al.* Adverse Pregnancy Outcomes and Sexual Violence Among Female Sex Workers Who Inject Drugs on the United States–Mexico Border. *Violence Vict* 2013;**28**:496–512. doi:10.1891/0886-6708.11-00129

29 Campbell MK, Piaggio G, Elbourne DR, *et al.* Consort 2010 statement: Extension to cluster randomised trials. *BMJ* 2012;**345**:19–23. doi:10.1136/bmj.e5661

30 Cheuk E, Isac S, Musyoki H, *et al.* Informing HIV prevention programs for adolescent girls and young women: A modified approach to programmatic mapping and key population size estimation. *J Med Internet Res* 2019;**21**:1–11. doi:10.2196/11196

For peer review only

Figure 1: Eligibility flow diagram for the WHISPER or SHOUT study as per Consort 2010 statement: extension to cluster randomized trials.[29]

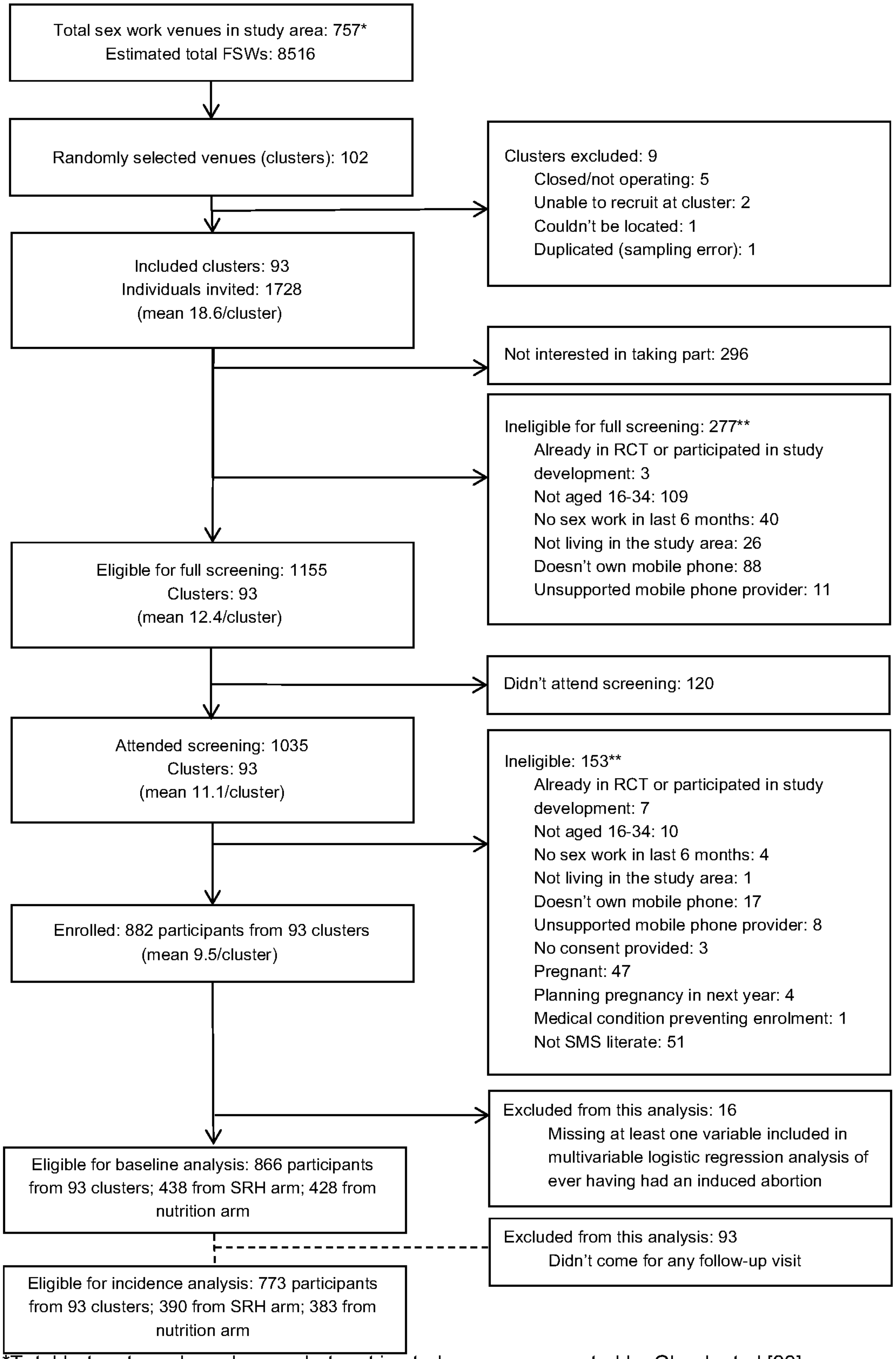

*Total hotspots and number per hotspot in study area enumerated by Cheuk et al.[30]

**One reason for ineligibility reported per participant and criteria determined in the order shown.

Figure 2: Overview of pregnancy outcomes during the 12-month follow-up. N=773

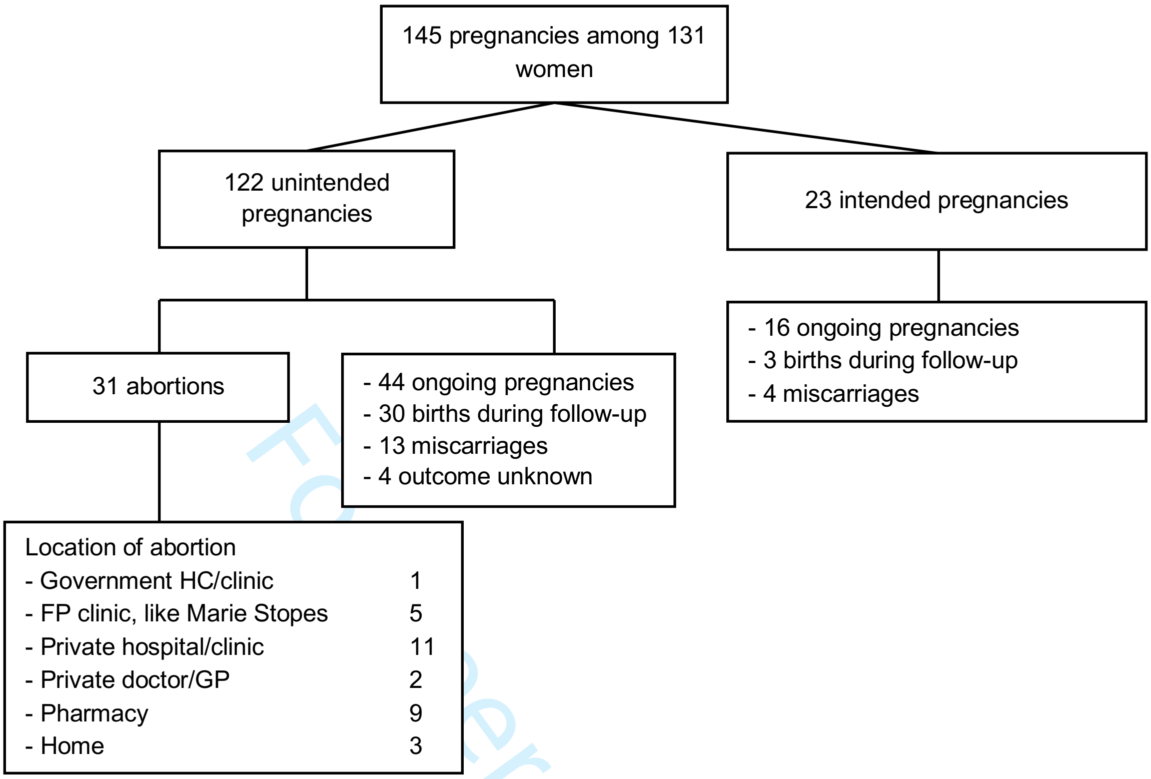

For peer review only

Table 1: CONSORT 2010 checklist of information to include when reporting a cluster randomised trial :

Protocol paper[1] and primary outcome paper[2]:

1 Ampt FH, Mudogo C, Gichangi P, *et al.* WHISPER or SHOUT study: Protocol of a cluster-randomised controlled trial assessing mHealth sexual reproductive health and nutrition interventions among female sex workers in Mombasa, Kenya. *BMJ Open* 2017;**7**. doi:10.1136/bmjopen-2017-017388

2 Ampt FH, Lim MSC, Agius PA, *et al.* Effect of a mobile phone intervention for female sex workers on unintended pregnancy in Kenya (WHISPER or SHOUT): a cluster-randomised controlled trial. *Lancet Glob Heal* 2020;**8**:e1534–45. doi:10.1016/S2214-109X(20)30389-2

Section/Topic	Item No	Standard Checklist item	Extension for cluster designs	Page No *
**Title and abstract**				
	1a	Identification as a randomised trial in the title	Identification as a cluster randomised trial in the title	**1**
	1b	Structured summary of trial design, methods, results, and conclusions (for specific guidance see CONSORT for abstracts)[1,2]	See table 2	**2**
**Introduction**				
**Background and objectives**	2a	Scientific background and explanation of rationale	Rationale for using a cluster design	**5 (and reference to protocol paper**[1]**)**
	2b	Specific objectives or hypotheses	Whether objectives pertain to the the cluster level, the individual participant level or both	**4 (and reference to protocol paper**[1]**)**
**Methods**				
**Trial design**	3a	Description of trial design (such as parallel, factorial) including allocation ratio	Definition of cluster and description of how the design features apply to the clusters	**5 (and reference to protocol paper**[1]**)**
	3b	Important changes to methods after trial commencement (such as eligibility criteria), with reasons		**N/A**

For peer review only - http://bmjopen.bmj.com/site/about/guidelines.xhtml

**Participants**	4a	Eligibility criteria for participants	Eligibility criteria for clusters	**5 (and reference to protocol paper**[1]**)**
	4b	Settings and locations where the data were collected		**5**
**Interventions**	5	The interventions for each group with sufficient details to allow replication, including how and when they were actually administered	Whether interventions pertain to the cluster level, the individual participant level or both	**5 (and reference to protocol paper**[1]**)**
**Outcomes**	6a	Completely defined pre-specified primary and secondary outcome measures, including how and when they were assessed	Whether outcome measures pertain to the cluster level, the individual participant level or both	**4, 6 (and reference to protocol paper**[1]**)**
	6b	Any changes to trial outcomes after the trial commenced, with reasons		**N/A**
**Sample size**	7a	How sample size was determined	Method of calculation, number of clusters(s) (and whether equal or unequal cluster sizes are assumed), cluster size, a coefficient of intracluster correlation (ICC or *k*), and an indication of its uncertainty	**Reference to protocol paper**[1]
	7b	When applicable, explanation of any interim analyses and stopping guidelines		**N/A**
**Randomisation:**				
**Sequence generation**	8a	Method used to generate the random allocation sequence		**5 (and reference to protocol paper**[1]**)**
	8b	Type of randomisation; details of any restriction (such as blocking and block size)	Details of stratification or matching if used	**Reference to protocol paper**[1]

For peer review only

**Allocation concealment mechanism**	9	Mechanism used to implement the random allocation sequence (such as sequentially numbered containers), describing any steps taken to conceal the sequence until interventions were assigned	Specification that allocation was based on clusters rather than individuals and whether allocation concealment (if any) was at the cluster level, the individual participant level or both	**Reference to protocol paper**[1]
**Implementation**	10	Who generated the random allocation sequence, who enrolled participants, and who assigned participants to interventions	Replace by 10a, 10b and 10c	**5 (and reference to protocol paper**[1]**)**
	10a		Who generated the random allocation sequence, who enrolled clusters, and who assigned clusters to interventions	**5 (and reference to protocol paper**[1]**)**
	10b		Mechanism by which individual participants were included in clusters for the purposes of the trial (such as complete enumeration, random sampling)	**5**
	10c		From whom consent was sought (representatives of the cluster, or individual cluster members, or both), and whether consent was sought before or after randomisation	**5**
**Blinding**	11a	If done, who was blinded after assignment to interventions (for example, participants, care providers, those assessing outcomes) and how		**Reference to protocol paper**[1]
	11b	If relevant, description of the similarity of interventions		**5 (and reference to protocol paper**[1]**)**
**Statistical methods**	12a	Statistical methods used to compare groups for primary and secondary outcomes	How clustering was taken into account	**6**

For peer review only

	12b	Methods for additional analyses, such as subgroup analyses and adjusted analyses		**6**
**Results**				
**Participant flow (a diagram is strongly recommended)**	13a	For each group, the numbers of participants who were randomly assigned, received intended treatment, and were analysed for the primary outcome	For each group, the numbers of clusters that were randomly assigned, received intended treatment, and were analysed for the primary outcome	**Figure 1**
	13b	For each group, losses and exclusions after randomisation, together with reasons	For each group, losses and exclusions for both clusters and individual cluster members	**Figure 1**
**Recruitment**	14a	Dates defining the periods of recruitment and follow-up		**8**
	14b	Why the trial ended or was stopped		**5, 8**
**Baseline data**	15	A table showing baseline demographic and clinical characteristics for each group	Baseline characteristics for the individual and cluster levels as applicable for each group	**8, 9**
**Numbers analysed**	16	For each group, number of participants (denominator) included in each analysis and whether the analysis was by original assigned groups	For each group, number of clusters included in each analysis	**Figure 1, Table 1, 2 and 3**
**Outcomes and estimation**	17a	For each primary and secondary outcome, results for each group, and the estimated effect size and its precision (such as 95% confidence interval)	Results at the individual or cluster level as applicable and a coefficient of intracluster correlation (ICC or k) for each primary outcome	**Table 2 and 3**
	17b	For binary outcomes, presentation of both absolute and relative effect sizes is recommended		**Table 2 and 3**
**Ancillary analyses**	18	Results of any other analyses performed,		**Table 2 and 3**

		including subgroup analyses and adjusted analyses, distinguishing pre-specified from exploratory		
**Harms**	19	All important harms or unintended effects in each group (for specific guidance see CONSORT for harms[3])		**Reference to primary outcome paper**[2]
**Discussion**				
**Limitations**	20	Trial limitations, addressing sources of potential bias, imprecision, and, if relevant, multiplicity of analyses		**13, 14**
**Generalisability**	21	Generalisability (external validity, applicability) of the trial findings	Generalisability to clusters and/or individual participants (as relevant)	**12, 13**
**Interpretation**	22	Interpretation consistent with results, balancing benefits and harms, and considering other relevant evidence		**12, 13**
**Other information**				
**Registration**	23	Registration number and name of trial registry		**15**
**Protocol**	24	Where the full trial protocol can be accessed, if available		**Reference to protocol paper**[1] **(page 5)**
**Funding**	25	Sources of funding and other support (such as supply of drugs), role of funders		**2**

** Note: page numbers optional depending on journal requirements*

For peer review only

Table 2: Extension of CONSORT for abstracts[1,2] to reports of cluster randomised trials

Item	Standard Checklist item	Extension for cluster trials
**Title**	Identification of study as randomised	**Identification of study as cluster randomised**
**Trial design**	Description of the trial design (e.g. parallel, cluster, non-inferiority)	
**Methods**		
**Participants**	Eligibility criteria for participants and the settings where the data were collected	**Eligibility criteria for clusters**
**Interventions**	Interventions intended for each group	
**Objective**	Specific objective or hypothesis	**Whether objective or hypothesis pertains to the cluster level, the individual participant level or both**
**Outcome**	Clearly defined primary outcome for this report	**Whether the primary outcome pertains to the cluster level, the individual participant level or both**
**Randomization**	How participants were allocated to interventions	**How clusters were allocated to interventions**
**Blinding (masking)**	Whether or not participants, care givers, and those assessing the outcomes were blinded to group assignment	
**Results**		
**Numbers randomized**	Number of participants randomized to each group	**Number of clusters randomized to each group**
**Recruitment**	Trial status[1]	
**Numbers analysed**	Number of participants analysed in each group	**Number of clusters analysed in each group**
**Outcome**	For the primary outcome, a result for each group and the estimated effect size and its precision	**Results at the cluster or individual participant level as applicable for each primary outcome**
**Harms**	Important adverse events or side effects	
**Conclusions**	General interpretation of the results	
**Trial registration**	Registration number and name of trial register	
**Funding**	Source of funding	

[1] Relevant to Conference Abstracts

REFERENCES

[1] Hopewell S, Clarke M, Moher D, Wager E, Middleton P, Altman DG, et al. CONSORT for reporting randomised trials in journal and conference abstracts. *Lancet* 2008, 371:281-283

[2] Hopewell S, Clarke M, Moher D, Wager E, Middleton P, Altman DG at al (2008) CONSORT for reporting randomized controlled trials in journal and conference abstracts: explanation and elaboration. *PLoS Med* 5(1): e20

[3] Ioannidis JP, Evans SJ, Gotzsche PC, O'Neill RT, Altman DG, Schulz K, Moher D. Better reporting of harms in randomized trials: an extension of the CONSORT statement. *Ann Intern Med* 2004; 141(10):781-788.

For peer review only

BMJ Open

ASSESSMENT OF THE LIFETIME PREVALENCE AND INCIDENCE OF INDUCED ABORTION AND CORRELATES AMONG FEMALE SEX WORKERS IN MOMBASA, KENYA: A SECONDARY COHORT ANALYSIS

Journal:	*BMJ Open*
Manuscript ID	bmjopen-2021-053218.R1
Article Type:	Original research
Date Submitted by the Author:	16-Jan-2022
Complete List of Authors:	Simmelink, Anne Marieke; The Aga Khan University - Kenya, Population Health<br>Gichuki, Caroline M.; The Aga Khan University Hospital Nairobi, Department of Population Health; International Centre for Reproductive Health Kenya<br>Ampt, Frances H.; Burnet Institute, Department of Epidemiology and Preventive Medicine; Monash University, Department of Epidemiology and Preventive Medicine<br>Manguro, Griffins; International Centre for Reproductive Health Kenya<br>Lim, Megan; Burnet Institute; Monash University, Department of Epidemiology and Preventive Medicine<br>Agius, Paul; Burnet Institute, Department of Epidemiology and Preventive Medicine; Monash University, Department of Epidemiology and Preventive Medicine<br>Hellard, Margaret; Burnet Institute, Department of Epidemiology and Preventive Medicine; Monash University, Department of Epidemiology and Preventive Medicine<br>Jaoko, Walter; University of Nairobi, Department of Medical Microbiology<br>Stoové, Mark; Burnet Institute, Department of Epidemiology and Preventive Medicine; Monash University, Department of Epidemiology and Preventive Medicine<br>L'Engle, Kelly; University of San Francisco<br>Temmerman, Marleen; International Centre for Reproductive Health Kenya; The Aga Khan University Hospital Nairobi, Department of Obstetrics and Gynaecology<br>Gichangi, Peter; International Centre for Reproductive Health Kenya; Ghent University, Department of Public Health and Primary Care<br>Luchters, Stanley; The Aga Khan University Hospital Nairobi, Department of Population Health; Burnet Institute, Department of Population Health
**Primary Subject Heading**:	Sexual health
Secondary Subject Heading:	Sexual health, Public health
Keywords:	SEXUAL MEDICINE, PUBLIC HEALTH, EPIDEMIOLOGY

For peer review only - http://bmjopen.bmj.com/site/about/guidelines.xhtml

I, the Submitting Author has the right to grant and does grant on behalf of all authors of the Work (as defined in the below author licence), an exclusive licence and/or a non-exclusive licence for contributions from authors who are: i) UK Crown employees; ii) where BMJ has agreed a CC-BY licence shall apply, and/or iii) in accordance with the terms applicable for US Federal Government officers or employees acting as part of their official duties; on a worldwide, perpetual, irrevocable, royalty-free basis to BMJ Publishing Group Ltd ("BMJ") its licensees and where the relevant Journal is co-owned by BMJ to the co-owners of the Journal, to publish the Work in this journal and any other BMJ products and to exploit all rights, as set out in our licence.

The Submitting Author accepts and understands that any supply made under these terms is made by BMJ to the Submitting Author unless you are acting as an employee on behalf of your employer or a postgraduate student of an affiliated institution which is paying any applicable article publishing charge ("APC") for Open Access articles. Where the Submitting Author wishes to make the Work available on an Open Access basis (and intends to pay the relevant APC), the terms of reuse of such Open Access shall be governed by a Creative Commons licence – details of these licences and which Creative Commons licence will apply to this Work are set out in our licence referred to above.

Other than as permitted in any relevant BMJ Author's Self Archiving Policies, I confirm this Work has not been accepted for publication elsewhere, is not being considered for publication elsewhere and does not duplicate material already published. I confirm all authors consent to publication of this Work and authorise the granting of this licence.

For peer review only

ASSESSMENT OF THE LIFETIME PREVALENCE AND INCIDENCE OF INDUCED ABORTION AND CORRELATES AMONG FEMALE SEX WORKERS IN MOMBASA, KENYA: A SECONDARY COHORT ANALYSIS

A. Marieke Simmelink[1], Caroline M. Gichuki[1,2], Frances H. Ampt[3,4], Griffins Manguro[2], Megan S.C. Lim[3,4], Paul A. Agius[3,4], Margaret Hellard[3,4,5,6], Walter Jaoko[7], Mark Stoové[3,4,8], Kelly L'Engle[9], Marleen Temmerman[2,10,11], Peter Gichangi[2,10,12], Stanley Luchters[1,3,4,10]

AFFILIATIONS

1. Department of Population Health, Medical College, Aga Khan University, Nairobi, Kenya
2. International Centre for Reproductive Health (ICRH), Mombasa, Kenya
3. Burnet Institute, Melbourne, Australia
4. Department of Epidemiology and Preventive Medicine, Monash University, Melbourne, Australia
5. Department of Infectious Diseases, The Alfred Hospital, Melbourne, Australia
6. Doherty Institute and School of Population and Global Health, University of Melbourne, Australia
7. Department of Medical Microbiology, University of Nairobi, Nairobi, Kenya
8. School of Psychology and Public Health, La Trobe University, Melbourne, Australia
9. University of San Francisco, San Francisco, California, USA
10. Department of Public Health and Primary Care, Ghent University, Ghent, Belgium
11. Department of Obstetrics and Gynaecology, Aga Khan University, Kenya
12. Technical University of Mombasa, Mombasa, Kenya

CORRESPONDING AUTHOR

Stanley Luchters, Chair of the Department of Population Health, Medical College, Aga Khan University, Nairobi, Kenya. Email: stanley.luchters@aku.edu

Word count: 2985

For peer review only - http://bmjopen.bmj.com/site/about/guidelines.xhtml

STRUCTURED ABSTRACT

Introduction

Prevalence of lifetime induced abortion in female sex workers (FSWs) in Kenya was previously estimated between 43 and 86%. Our analysis aimed at assessing lifetime prevalence and correlates, and incidence and predictors of induced abortions among FSWs in Kenya.

Methods

This is a secondary prospective cohort analysis using data collected as part of the WHISPER or SHOUT cluster-randomized trial in Mombasa (Australian New Zealand Clinical Trials Registry number: ACTRN12616000852459), assessing effectiveness of an SMS-intervention to reduce incidence of unintended pregnancy. Eligible participants were current FSWs, 16-34 years and not pregnant or planning pregnancy. Baseline data on self-reported lifetime abortion, correlates and predictors were collected between September 2016 and May 2017. Abortion incidence was measured at six- and twelve-months follow-up. A multivariable logistic regression model was used to assess correlates of lifetime abortion and discrete-time survival analysis was used to assess predictors of abortions during follow-up.

Results

Among 866 eligible participants, lifetime abortion prevalence was 11.9%, while lifetime unintended pregnancy prevalence was 51.2%. Correlates of lifetime abortions were currently not using a highly effective contraceptive (AOR=1.76 [95%CI=1.11-2.79] p=0.017) and having ever experienced intimate partner violence (IPV) (AOR=2.61 [95%CI=1.35-5.06] p=0.005). Incidence of unintended pregnancy and induced abortion were 15.5 and 3.9 per 100 women-years, respectively. No statistically significant associations were found between hazard of abortion and age, sex work duration, partner status, contraceptive use and IPV experience.

Conclusion

Although experience of unintended pregnancy remains high, lifetime prevalence of abortion may have decreased among FSW in Kenya. Addressing IPV could further decrease induced abortions in this population.

KEYWORDS

Sex Work, induced abortion, unintended pregnancy, contraception, cluster-random sample, Kenya

FUNDING STATEMENT

66 This work was supported by Australia's National Health and Medical Research Council
67 (NHMRC), Project Grant GNT 1087006.

For peer review only

ARTICLE SUMMARY

Strengths and limitations

- This study presents incidence of abortion in a cluster-randomised cohort of FSWs.
- It is the first to analyse predictors of abortions in FSWs, rather than correlates of past abortions only.
- This paper explores a secondary research question, and the study was not originally powered to assess the predictors of abortions during follow-up.
- The sensitive topic of abortions and SRH in general, might have resulted in a social desirability bias.

For peer review only

For peer review only - http://bmjopen.bmj.com/site/about/guidelines.xhtml

INTRODUCTION

Research findings show that about 5% of Kenya's urban female reproductive population could be involved in sex work.[1] Female sex workers (FSWs) experience higher than average rates of HIV, other sexually transmitted infections (STIs) and unintended pregnancy.[2–5]

Unintended pregnancies often have negative consequences for FSWs, including financial adversity, social stigma and induced abortion.[4] In countries where abortion is illegal or difficult to access, women frequently resort to unsafe practices, risking severe medical complications.[6]

Reported prevalence of lifetime abortion among FSWs in low- and middle-income countries (LMICs) varies from 24% in Laos in 2012 to 86% in 2000/01 in Central and Western Kenya.[7–16] Many of these abortions are unsafe or sought in the informal sector.[8,11,16] In Mombasa, lifetime abortion among FSWs was estimated at 43% in 2008.[17]

In 2010, Kenya liberalized its abortion law, making abortion legal when "there is need for emergency treatment, or the life or health of the mother is in danger".[18] In practice, older laws criminalizing abortion remain in place, creating ambiguity among health professionals with lawsuits remaining a threat.[19] Moreover, social, cultural and religious beliefs condemning pregnancy termination, misconceptions about the illegality and costs of the procedure, still hamper access to safe services for women.[4,19]

A national study in 2012 estimated an induced abortion rate of 48 per 1,000 women among women aged 15-49, based on data of women who sought care for abortion complications.[20] It showed a diverse sociodemographic and economic background among these women in terms of educational level, employment status, marital status and religion.

Incidence of induced abortions among FSWs has not been studied in Kenya and studies analysing correlates of induced abortions elsewhere have been cross-sectional and report correlates of lifetime abortions, precluding attribution of causality.[8–15,21] Examining incidence of abortions and identifying predictors will help inform future policies to improve care around abortions for FSWs and the need for longitudinal data about abortions in FSWs has therefore been recognised.[8,14]

This secondary data analysis aimed to examine lifetime prevalence and correlates, and incidence and predictors of induced abortions in a cluster-random sample of FSWs in Mombasa, Kenya.

For peer review only - http://bmjopen.bmj.com/site/about/guidelines.xhtml

MATERIAL AND METHODS

Study design

This is a secondary prospective cohort analysis using data collected as part of the WHISPER or SHOUT cluster-randomized trial in Mombasa. A detailed description of the study protocol can be found elsewhere.[22] In summary, the study was a two-arm cluster-randomized controlled trial assessing the effectiveness of two SMS-based interventions targeting sexual and reproductive health (SRH) and nutrition in FSWs in Mombasa, Kenya. The study was conducted in two sub-counties in Mombasa, Kisauni and Changamwe, between September 2016 and July 2018. Ninety-three venues were randomly sampled with a probability proportional to FSW population size at the venue (Figure 1). Trained community mobilisers and peer educators recruited FSWs from the venues until the required sample size of 860 FSWs was achieved. This study uses baseline and follow-up data from the trial. During the recruitment period of 14 September 2016 to 16 May 2017, 882 women were enrolled in the study. Follow-up continued until 31 July 2018. A sub-sample of 866 women (98.2%) was analysed for this secondary analysis (Figure 1).

Study participants and procedures

Women were eligible for the study if they were aged 16-34 years; self-reported to have engaged in paid sex work in the last 6 months; were reportedly not pregnant or planning pregnancy within the next 12 months; resided within the study area; and were able to read text messages in basic English. Study-specific community mobilizers visited the selected clusters to recruit FSWs and conducted pre-screening interviews identifying women who self-reported to be sex workers. Potentially eligible FSWs were referred to the nearest study clinic for a clinical assessment, including a urine pregnancy test, and STI and HIV testing. Enrolled participants then completed a structured questionnaire administered in Swahili by trained research assistants, who had previously participated in research with FSWs. The questionnaire captured detailed sociodemographic, sexual and reproductive health information. Follow-up visits were scheduled at 6 and 12 months after enrolment. Procedures at follow-up visits were similar to those done at enrolment.
Participants received two to three SMS messages per week for 12 months. The messages consisted of stand-alone push messages, role model stories and on-demand messages, accessed using assigned codes. Participants only received and accessed messages on their phones from their assigned intervention.

Outcomes and correlates

Lifetime prevalence of induced abortion was assessed at baseline with the question ‘How many times have you ever had an induced abortion?’. Induced abortions were assessed

For peer review only

during follow-up, by asking participants if they had been pregnant since their last visit. The outcome of each reported pregnancy was then assessed, and in the case of an induced abortion, the location was documented. Formal sector abortions were those taking place at a government or private hospital/clinic, a private doctor/General Practitioner (GP) or a Family Planning (FP) clinic. Informal sector abortions were defined as those taking place at home, a pharmacy or traditional healer.[23]

Pregnancies during follow-up were confirmed with a urine pregnancy test at the study clinic, or self-reported by the participant when occurring between study visits. Pregnancy intention for all reported pregnancies was assessed using the London Measure of Unintended Pregnancy (LMUP), a six-item scale. A pregnancy scoring less than 10 out of 12 on the LMUP was defined as unintended.[24]

All correlates of lifetime induced abortions and predictors of incident induced abortions were self-reported at baseline. Use of a highly effective contraceptive method was defined as use of contraceptive implants, IUD, injection, oral contraceptive pill and sterilization. High knowledge on FP was defined as answering five out of six true-false statements on FP correctly. Having a positive attitude on FP was defined as agreeing with at least three out of four positive attitude statements. Self-efficacy in FP was defined as high when agreeing with two statements on this topic. Household socio-economic status tertiles were generated using principal component analysis, based on 12 household assets.[25] Prevalence of intimate partner violence (IPV) was assessed by asking if the participant had ever been pushed, slapped, hit or kicked by a partner, or had been physically forced to have sex, agreed to sex out of fear of the consequences or forced to do something sexual that she perceived as degrading or humiliating. For each item, it was also assessed if this happened in the previous 12 months.

Statistical analysis

Statistical analysis was performed using Stata software version 14 (StataCorp, College Station, TX, USA). Distributions of socio-demographic characteristics, reproductive history, contraceptive use and intimate partner violence at baseline were explored with means and standard deviations for continuous variables and proportions for categorical variables. Covariates age, education level, SES-tertile, duration of sex work, having a husband/boyfriend, use of highly effective contraceptives and experience of IPV, were included in the multivariate models on the basis of a review of literature.[8–15,21] Covariates high FP knowledge, positive attitude to FP and high FP specific self-efficacy were included in the multivariate models on the basis of theoretical assumptions by the co-authors. It was hypothesised that these characteristics would be positively associated with contraceptive use and would protect against experiencing an induced abortion. Correlates of lifetime

For peer review only - http://bmjopen.bmj.com/site/about/guidelines.xhtml

abortion were identified using weighted multivariable logistic regression. Associations were considered statistically significant at the 5% level. The outcome incident abortion was interval-censored (measured at 6-monthly intervals). Therefore, a discrete-time survival analysis was performed using generalized linear mixed (GLM) modelling with complementary log-log link function and binomial distribution, a method that produces estimated hazard ratios. Abortions during follow-up were analysed for all participants who attended at least one follow-up visit. All outcomes presented here are cluster-adjusted, based on inverse probability sample means to account for sampling bias.

Ethical consideration

All study participants provided written informed consent. The study was approved by the Kenyatta National Hospital and University of Nairobi Ethics and Research Committee, Kenya (KNH-UoN ERC—KNH-ERC/RR/493) and the Monash University Human Research Ethics Committee, Australia (MUHREC—CF16/1552—2016000812).

Patient and public involvement

Patients or the public were not directly involved in the design, or conduct, or reporting, or dissemination plans of our research.

For peer review only

RESULTS

Mean age was 25.5 years (SD=4.7) (Table 1). The majority of women (n=765, 88.7%) had at least completed primary education and 306 (34.9%) had completed secondary education. Just over half of the participants (n=484, 56.6%) reported to have a current husband or boyfriend, but 812 (94.0%) reported not to live with a partner. Mean duration of employment in sex work was 4.7 years (SD=3.5). Among women currently reporting a husband or boyfriend, 344/483 (70.8%) had not disclosed their employment in sex work to their partners. 605/861 women (68.9%) worked fulltime in sex work and 508/863 (59.8%) earned more than 2000 Ksh (about USD $20) per week from sex work. The majority of women (n=666, 76.1%) had ever been pregnant, and 451 (51.2%) ever had an unintended pregnancy. 103/866 (11.9%) reported to have had at least one induced abortion in their lifetime. Among women who had an induced abortion, 58/102 (57.1%) went to a private hospital or clinic for the most recent abortion. 29/102 (29.1%) women had their most recent abortion in the informal sector, like home, a pharmacy or traditional healer. At baseline, 463 women (54.4%) reported to use a highly effective contraceptive method. Three-quarters of FSW (650/866; 75.0%) ever experienced IPV and 525/866 (60.1%) experienced IPV in the past 12 months the before the baseline questionnaire was conducted.

Table 1. Socio-demographic characteristics, reproductive history, contraceptive use and intimate partner violence at baseline of female sex workers in Mombasa, Kenya (n=866, unless stated otherwise)

Characteristic	n	Cluster-adjusted mean (SD) or proportion in % (95% CI)[a]
Mean age, in years		25.5 (4.7)
Highest level of education		
None or some primary	101	11.2 (9.2-13.6)
Completed primary or some secondary	459	53.8 (50.1-57.6)
Completed secondary or some tertiary	306	34.9 (31.3-38.7)
Religion (n=864)		
Protestant	389	44.8 (41.4-48.2)
Catholic	304	36.0 (32.3-39.8)
Muslim	171	19.2 (15.6-23.4)
Electricity available in household (n=863)[b]	660	76.4 (73.0-79.5)
Duration of sex work, in years		4.7 (3.5)
Fulltime FSW (n=861)[c]	605	68.9 (64.0-73.5)
Weekly income from sex work (n=861)		
≤1000 Ksh	144	16.2 (13.2-19.8)
1001-2000 Ksh	211	24.0 (21.0-27.3)

For peer review only - http://bmjopen.bmj.com/site/about/guidelines.xhtml

≥2001 Ksh[d]	508	59.8 (55.1-64.3)
Sex work venue		
Bar with lodging	388	43.8 (37.7-50.2)
Bar without lodging	147	17.2 (13.7-21.3)
Lodging/guesthouse	138	15.1 (10.9-20.5)
Street/beach	86	11.2 (7.8-15.7)
Other[e]	107	12.7 (9.1-17.5)
Marital status		
Married/cohabiting	54	6.0 (4.5-8.0)
Single (not cohabiting)	627	73.0 (69.6-76.2)
Separated/divorced/widowed	185	21.0 (18.0-24.3)
Currently has husband/boyfriend	484	56.6 (52.5-60.7)
Disclosure of sex work to husband/boyfriend (n=483)[f]		
Yes	136	28.7 (24.4-33.3)
No	344	70.8 (66.0-75.1)
Don't know	3	0.6 (0.2-1.7)
Ever had a pregnancy	666	76.1 (72.3-79.5)
Has a living child	622	71.2 (67.1-75.0)
Ever had an unintended pregnancy (N=864)[g]	451	51.2 (47.4-54.9)
Ever had an induced abortion	103	11.9 (10.0-14.2)
Location of most recent induced abortion (N=102)		
Government hospital	2	1.8 (0.4-6.9)
FP clinic, like Marie Stopes	9	8.3 (4.4-15.0)
Private hospital/clinic	58	57.1 (46.5-67.2)
Private doctor GP	4	3.7 (1.4-9.6)
Pharmacy	9	8.9 (4.6-16.8)
Traditional healer	4	3.8 (1.4-10.0)
Home	16	16.4 (9.0-27.8)
Uses a highly effective contraceptive method[h]	473	54.4 (49.5-59.2)
Ever experienced IPV[i]	650	75.0 (71.1-78.5)
Experienced IPV in past 12 months[j]	525	60.1 (55.5-64.6)

[a] Inverse probability-weighted percentages. [b] Availability of electricity in the household is presented here as a proxy for household SES. [c] Fulltime work as FSW is characterized as having no other sources of income in the last 6 months. [d] 1000 Kenyan Shilling (Ksh) is about USD $10. [e] Brothel, strip club, casino, massage parlors, parks or home. [f] Among participants with a husband or boyfriend. [g] Assessed using the London Measure of Unintended Pregnancy (LMUP) [h] Highly effective is defined as use of contraceptive implants, IUD, injection, oral contraceptive pill and sterilization. [i] IPV= intimate partner violence. [j] Before baseline.

Women currently not using a highly effective contraceptive (AOR=1.76 [95%CI=1.11-2.79] p=0.017) and women who ever experienced IPV (AOR=2.61 [95%CI=1.35-5.06] p=0.005) were significantly more likely to report a history of induced abortion, when controlled for potential confounders (Table 2). Longer duration of sex work showed a borderline positive association with history of abortion (AOR=1.08 [95%CI=1.00-1.16] p=0.053). Although

For peer review only - http://bmjopen.bmj.com/site/about/guidelines.xhtml

higher age was significantly associated with a history of abortion in bivariate analysis, after adjusting for confounding factors this association was no longer seen.

Table 2. Correlates of participants with a history of induced abortion, and cluster-adjusted bi- and multivariable logistic regression analysis on history of induced abortion (n=866)

Characteristic		Crude Odds Ratio (OR)		Adjusted OR	
	Ever had an induced abortion (n=103); n/N (cluster-adjusted proportion in %)[a]	OR (95% CI)[b]	p-value	OR (95% CI)[b]	p-value
Age (in years)	27.0 (4.9)[c]	1.08 (1.03-1.14)	**0.001**	1.04 (0.97-1.11)	0.280
Highest level of education					
None or some primary	14/101 (13.5)	Ref.		Ref.	
Completed primary or some secondary	57/459 (12.4)	0.90 (0.47-1.72)	0.749	0.96 (0.48-1.99)	0.895
Completed secondary or some tertiary	32/306 (10.7)	0.77 (0.40-1.47)	0.423	0.83 (0.40-1.84)	0.620
SES-tertile[e]					
Poorest	39/290 (13.4)	Ref.		Ref.	
Middle	33/287 (10.9)	0.79 (0.46-1.35)	0.386	0.89 (0.50-1.59)	0.697
Richest	31/289 (11.4)	0.83 (0.47-1.46)	0.510	0.83 (0.46-1.50)	0.674
Mean duration of sex work (in years)	6.1 (3.4)[c]	1.12 (1.07-1.17)	**<0.001**	1.08 (1.00-1.16)	0.053
Highly effective contraceptive use[d]					
Yes	51/473 (10.6)	Ref.		Ref.	
No	52/393 (13.5)	1.32 (0.88-1.97)	0.173	1.76 (1.11-2.79)	**0.017**
High FP knowledge score					
No	60/562 (10.5)	Ref.		Ref.	
Yes	43/304 (14.5)	1.44 (0.95-2.19)	0.084	1.34 (0.85-2.10)	0.200
Positive attitude to FP use					
No	43/354 (12.4)	Ref.		Ref.	
Yes	60/512 (11.6)	0.93 (0.60-1.44)	0.743	0.90 (0.56-1.45)	0.661
High FP-specific self-efficacy					
No	24/237 (10.1)	Ref.		Ref.	
Yes	79/629 (12.6)	1.28 (0.76-2.17)	0.345	1.23 (0.72-2.10)	0.454
Ever experienced intimate partner violence					
No	12/216 (5.2)	Ref.		Ref.	
Yes	91/650 (14.1)	2.98 (1.55-5.74)	**0.001**	2.61 (1.35-5.06)	**0.005**

[a] Inverse probability-weighted percentages. [b] Standard errors are corrected by cluster sandwich variance estimation. [c] Mean (SD) of women who ever had an induced abortion. [d] Highly effective is defined as use of contraceptive implants, IUD, injection, oral contraceptive pill and sterilization. [e] SES = Socio-economic status.

During the study follow-up, 773 women attended at least one follow-up visit (Figure 1). Total follow-up time was 9,468 months, with an average of 12.2 months per woman (data not shown). A total of 131 participants became pregnant, with a total of 145 pregnancies among these women (Figure 2). Of these pregnancies, 122/145 were unintended according to the LMUP. Among 145 pregnancies, 31 ended in induced abortion, among 29 women and

For peer review only - http://bmjopen.bmj.com/site/about/guidelines.xhtml

across 789 women-years at risk. Overall incidence rate was 3.9 induced abortions per 100 women-years of observation. Out of 31 abortions, 19 took place in the formal sector and 12 in an informal setting.

The GLM modelling of abortion incidence showed that women experiencing IPV in the past year (HR=1.93 [95%CI=0.86-4.34] p=0.122) and women not using a highly effective contraceptive (HR=1.51 [95%CI=0.66-3.49] p=0.332) exhibited a higher hazard of abortion, independent of other factors, although these results were not significant (Table 3). We did not find a relation between age, mean duration of sex work, currently having a husband or boyfriend and the intervention under study and hazard of induced abortion.

Table 3. Baseline predictors of incident abortion in FSWs among Mombasa, Kenya (N=773)[a]

Baseline predictors of incident abortions	Unadjusted[b]		Adjusted HR[c]	
	**HR (95% CI)[d]**	**p-value**	**HR (95% CI)[d]**	**p-value**
Age (in years)	0.96 (0.89-1.04)	0.315	1.00 (0.91-1.09)	0.918
Mean duration of sex work (in years)	0.93 (0.82-1.05)	0.234	0.92 (0.79-1.09)	0.336
Currently has husband/boyfriend	0.83 (0.40-1.73)	0.622	0.80 (0.39-1.64)	0.537
Not using highly effective contraceptive[e]	1.50 (0.69-3.23)	0.310	1.51 (0.66-3.49)	0.332
Experienced IPV in last 12 months	1.67 (0.74-3.79)	0.216	1.93 (0.86-4.34)	0.122

[a] Discrete-time survival analysis including the first induced abortion per women. Generalized linear mixed model with complementary log-log link, binomial distribution, offset for log time between visits and random intercept for participants. [b] HR = Hazard Ratio. [c] All adjusted Hazard Ratios are also adjusted for the intervention. The intervention had no detectable effect on the outcome of incident abortions. [d] Cluster robust standard errors for sex-work venue clustering. [e] Highly effective is defined as use of contraceptive implants, IUD, injection, oral contraceptive pill and sterilization.

For peer review only

DISCUSSION

This study adds to the current knowledge of abortion practices in FSWs. Lifetime induced abortion prevalence in this population was 11.9%. This seems considerably lower than previous figures of lifetime abortion of 86% in 2004 in central and western Kenya among FSWs of a similar age, and 43% in 2008 in Mombasa among FSWs who were on average 2 years older.[7,17] In the former study, it was not specified if these abortions also included spontaneous abortions, which might have overestimated the prevalence of abortions. However, despite the sociodemographic and methodological differences between the studies, the size of the difference is suggestive of an actual lower rate in abortions in this population. The prevalence of abortions found here is also lower than reports from other LMICs ranging between 24% and 64% in Laos and Cote d'Ivoire, respectively.[8–16] A possible explanation for this lower prevalence is the relatively high use of highly effective contraceptives of 54% in our cohort, compared to similar studies from LMICs. [8,12,14–16] The findings are furthermore consistent with a lower-than-expected HIV prevalence and unintended pregnancy incidence in our cohort and could be a result of peer-mediated interventions implemented over the past years in the Mombasa area.[26,27] These have mostly targeted prevention of HIV and STIs, but likely have had a lowering effect on unintended pregnancies and induced abortions as well.[22,26] Furthermore, this study, in contrast to above referenced studies, attempted to draw a representative sample of a FSW population from community settings, whereas other studies used non-probability sampling methods, which might have overestimated past abortions.

Despite the lower-than-expected unintended pregnancy incidence, still 51% of FSWs in our cohort reported an unintended pregnancy in their lifetime. The gap between lifetime unintended pregnancies and lifetime induced abortions could indicate that many women decide to keep a child from unintended pregnancies, which could be supported by the fact that between 70-80% of young FSW in Mombasa have reported one or more children.[4,27] It may also indicate a high unmet need for induced abortion services among FSWs, for example due to ongoing or increasing difficulties in accessing SRH or abortion services for this group or increasing sociocultural barriers to abortion. Barriers to accessing other SRH services such as long-acting reversible contraceptives has previously been reported for this population.[28]

The present study is one of the few studies to report incidence of abortion among FSWs and to our knowledge, the first to analyse predictors of abortions in FSWs, rather than correlates of past abortions only. Incidence of induced abortion in our cohort was 3.9 per 100 women-years. Compared to other studies from LMICs, this is similar to two studies reporting abortion incidence rates of 3.1 and 3.0 per 100 women-years and lower than a third study reporting 7.4 induced abortions per 100 women-years among FSWs.[29] The intervention

For peer review only - http://bmjopen.bmj.com/site/about/guidelines.xhtml

under study had no measurable effect on unintended pregnancy incidence and is therefore unlikely to have affected incidence of induced abortions.[27]

Informal sector abortions where common in this cohort, with 29% of women having had their most recent abortion in the informal sector, and 39% of the reported abortions during follow-up happening in the informal sector. These informal sector abortions, put women at higher risk of complications due to unsafe practices and this denotes a need for information on safer alternatives, like the Marie Stopes clinics.[23]

Multiple studies have found both age and duration of sex work to be correlated to past abortions. Commonly higher age [8,11,15] and longer duration [9,12] of sex work were associated with higher lifetime abortion prevalence. One study found that younger age was associated with past abortions.[10] In our cohort, although FSWs with a past abortion in our cohort were older in the crude analysis, after adjusting for other correlates, this difference was no longer significant. The association with longer duration of sex work remained borderline significant in multivariate analysis. We did not find a relation between age and mean duration of sex work and having an induced abortion during follow-up. This might suggest that the association between past abortions and higher age and longer duration of sex work is caused by cumulative exposure to high risk of pregnancies and abortion.

We found a positive association between currently not using a highly effective contraceptive and having a past abortion. No difference was found in FP-specific self-efficacy or knowledge, or attitude towards FP among women with and without a past abortion. The found association could indicate significant barriers to uptake or continuation of a highly effective contraceptive method post abortion, as has previously been acknowledged by a study in Kenya.[30]

In our cohort, experience of IPV was high and the odds of having had a past abortion were more than 2.5-times as high for women who experienced IPV in the past, consistent with findings from other studies.[14,15] Our study also showed a positive association between experience of IPV in the past twelve months and abortions during follow-up, but this was not significant. Experience of (intimate partner) violence has been shown to have a negative effect on the reproductive health of FSWs, with greater risks of adverse pregnancy outcomes and forced termination of pregnancy.[31,32] Addressing the problem of IPV in this population could further lower induced abortions.

Limitations

Some limitations should be considered when interpreting the findings from this study. The sensitive topic of abortions and SRH in general, might have resulted in a social desirability bias. To minimize this, peer-educators and research assistants had previous experience working with the target population and received additional training. Attrition bias might have

For peer review only - http://bmjopen.bmj.com/site/about/guidelines.xhtml

occurred due to loss to follow-up of pregnant participants, as has been recognized by anecdotal evidence.[27] This might have resulted in an underestimation of abortions in our study. A further limitation is that this paper explores a secondary research question, and the study was not originally powered to assess the predictors of abortions during follow-up. Unknown timing of the past abortions in relation to studied correlates, restrict judgement of temporality of the studied associations. Lastly, measurements of abortions stopped when the intervention stopped, so the actual number of abortions during follow-up might in fact be higher than captured in the study.

Suggestions for further research

Future research is needed to explore the trend in abortion incidence among FSWs in Kenya. In order to improve care, we need to better understand current abortion practices, the decision-making process around terminating unintended pregnancies, how uptake of highly effective contraceptives can be increased post-abortion, as well as the relationship between experience of IPV and induced abortions.

CONCLUSIONS

In conclusion, the prevalence of lifetime induced abortions in a random cohort of FSWs in Mombasa, was 11.9% and incidence was 3.9 per 100 women-years, whereas prevalence and incidence of unintended pregnancies were higher at 51% and 15.5 per 100 women-years, respectively. A history of induced abortion was positively associated with not using a highly contraceptive method at baseline and having experienced IPV in the past. The study did not find a significant association with the studied predictors of abortions.

For peer review only - http://bmjopen.bmj.com/site/about/guidelines.xhtml

ACKNOWLEDGEMENTS

The authors would like to acknowledge the hard work of the field research team, specifically Christine Maghanga, Millicent Okello, Betty Kitili, Judith Wamaua, Elizabeth Bilasi, Marion Mwangi, Promillah Muindi, Rukia Abdallah and Khadija Kassim, as well as all the study participants of the study. The authors gratefully acknowledge the contribution of funding from Australia's National Health and Medical Research Council for the WHISPER or SHOUT trial (Project Grant GNT 1087006), Career Development Fellowships for SL, Senior Research Fellowship for MS, and a Postgraduate Scholarship for FHA. The sponsor did not contribute to study design; data collection, analysis, or interpretation; manuscript writing; or the decision to submit the article for publication. Lastly, we acknowledge the contribution of funding from the Victorian Operational Infrastructure Support Program received by the Burnet Institute.

TRIAL REGISTRATION

The trial was registered in the Australian New Zealand Clinical Trials Registry, ACTRN12616000852459.

AUTHOR CONTRIBUTORSHIP

SL was the Principal Investigator on the study. SL, FHA, PG, MSCL, PAA, MH, WJ, MS, KL, MT and MFC contributed to the study design. CG and GM coordinated the trial and undertook data acquisition in Kenya under the supervision of PG. AMS and SL conceptualized the manuscript. AMS and PAA conducted the statistical analyses. AMS and CG wrote the first draft of the manuscript. All authors contributed to data interpretation, provided critical input, and approved the final version of the manuscript.

DECLARATION OF INTERESTS

The authors have no conflicts of interest.

DATA AVAILABILITY STATEMENT

The original data are not available in a public repository. The corresponding author is to be contacted for the consideration of any data requests.

For peer review only

For peer review only - http://bmjopen.bmj.com/site/about/guidelines.xhtml

388 **FIGURE CAPTIONS**

389 **Figure 1:** Eligibility flow diagram for the WHISPER or SHOUT study as per Consort 2010
390 statement: extension to cluster randomized trials.[33]

391 **Figure 2:** Overview of pregnancy outcomes during the 12-month follow-up. N=773

For peer review only

REFERENCES

1 Odek WO, Githuka GN, Avery L, *et al.* Estimating the size of the female sex worker population in Kenya to inform HIV prevention programming. *PLoS One* 2014;**9**. doi:10.1371/journal.pone.0089180

2 Baral S, Beyrer C, Muessig K, *et al.* Burden of HIV among female sex workers in low-income and middle-income countries: A systematic review and meta-analysis. *Lancet Infect Dis* 2012;**12**:538–49. doi:10.1016/S1473-3099(12)70066-X

3 Cwikel JG, Lazer T, Press F, *et al.* Sexually transmissible infections among female sex workers: An international review with an emphasis on hard-to-access populations. *Sex Health* 2008;**5**:9–16. doi:10.1071/SH07024

4 Luchters S, Bosire W, Feng A, *et al.* 'A baby was an added burden': Predictors and consequences of unintended pregnancies for female sex workers in Mombasa, Kenya: A mixed-methods study. *PLoS One* 2016;**11**:1–20. doi:10.1371/journal.pone.0162871

5 Ippoliti NB, Nanda G, Wilcher R. Meeting the Reproductive Health Needs of Female Key Populations Affected by HIV in Low- and Middle-Income Countries: A Review of the Evidence. *Stud Fam Plann* 2017;**48**:121–51. doi:10.1111/sifp.12020

6 World Health Organization. WHO | Unsafe abortion: global and regional estimates of the incidence of unsafe abortion and associated mortality in 2008. 2014. doi:10.1017/CBO9781107415324.004

7 Elmore-Meegan M, Conroy RM, Agala CB. Sex Workers in Kenya, Numbers of Clients and Associated Risks: An Exploratory Survey. *Reprod Health Matters* 2004;**12**:50–7. doi:10.1016/S0968-8080(04)23125-1

8 Schwartz S, Papworth E, Thiam-Niangoin M, *et al.* An urgent need for integration of family planning services into HIV care: The high burden of unplanned pregnancy, termination of pregnancy, and limited contraception use among female sex workers in Côte d'Ivoire. *J Acquir Immune Defic Syndr* 2015;**68**:S91–8. doi:10.1097/QAI.0000000000000448

9 Bautista CT, Mejía A, Leal L, *et al.* Prevalence of lifetime abortion and methods of contraception among female sex workers in Bogota, Colombia. *Contraception* 2008;**77**:209–13. doi:10.1016/j.contraception.2007.11.013

10 Chanda MM, Ortblad KF, Mwale M, *et al.* Contraceptive use and unplanned pregnancy among female sex workers in Zambia. *Contraception* 2017;**96**:196–202. doi:10.1016/j.contraception.2017.07.003

11 Lau JTF, Mui LWH, Tsui HY, *et al.* Prevalence of induced abortion and associated factors among Chinese female sex workers in Hong Kong. *J Sex Marital Ther* 2007;**33**:19–29. doi:10.1080/00926230600998441

12 Todd CS, Alibayeva G, Sanchez JL, *et al.* Utilization of contraception and abortion and its relationship to HIV infection among female sex workers in Tashkent, Uzbekistan. *Contraception* 2006;**74**:318–23. doi:10.1016/j.contraception.2006.04.006

13 Zhang XD, Kennedy E, Temmerman M, *et al.* High rates of abortion and low levels of contraceptive use among adolescent female sex workers in Kunming, China: A cross-sectional analysis. *Eur J Contracept Reprod Heal Care* 2014;**19**:368–78. doi:10.3109/13625187.2014.927421

14 Zhang XD, Myers S, Yang HJ, *et al.* Prevalence and correlates of sexual and gender-based violence against Chinese adolescent women who are involved in commercial sex: A cross-sectional study. *BMJ Open* 2016;**6**. doi:10.1136/bmjopen-2016-013409

15 Karamouzian M, Mirzazadeh A, Shokoohi M, *et al.* Lifetime abortion of female sex workers in Iran: Findings of a national bio-behavioural survey In 2010. *PLoS One* 2016;**11**:1–12. doi:10.1371/journal.pone.0166042

16 Cleeve A, Phrasisombath K, Sychareun V, *et al.* Attitudes and experiences regarding induced abortion among female sex workers, Savannakhet Province, Laos. *Sex Reprod Healthc* 2014;**5**:137–41. doi:10.1016/j.srhc.2014.03.001

For peer review only - http://bmjopen.bmj.com/site/about/guidelines.xhtml

17 Sutherland EG, Alaii J, Tsui S, *et al.* Contraceptive needs of female sex workers in Kenya - A cross-sectional study. *Eur J Contracept Reprod Heal Care* 2011;**16**:173–82. doi:10.3109/13625187.2011.564683

18 Constitution of Kenya. Laws of kenya. *Natl Counc Law Report* 2010;:191. doi:10.1364/OE.17.019075\r186571 [pii]

19 Ushie BA, Juma K, Kimemia G, *et al.* Community perception of abortion, women who abort and abortifacients in Kisumu and Nairobi counties, Kenya. *PLoS One* 2019;**14**:1–13. doi:10.1371/journal.pone.0226120

20 Ministry of Health Republic of Kenya. Incidence and Complications of Unsafe Abortion in Kenya - Key Findings of a National Study. 2013.

21 Yi S, Tuot S, Chhoun P, *et al.* Factors associated with induced abortion among female entertainment workers: A cross-sectional study in Cambodia. *BMJ Open* 2015;**5**:1–8. doi:10.1136/bmjopen-2015-007947

22 Ampt FH, Mudogo C, Gichangi P, *et al.* WHISPER or SHOUT study: Protocol of a cluster-randomised controlled trial assessing mHealth sexual reproductive health and nutrition interventions among female sex workers in Mombasa, Kenya. *BMJ Open* 2017;**7**. doi:10.1136/bmjopen-2017-017388

23 Chemlal S, Russo G. Why do they take the risk? A systematic review of the qualitative literature on informal sector abortions in settings where abortion is legal. *BMC Womens Health* 2019;**19**:1–11. doi:10.1186/s12905-019-0751-0

24 Hall J, Barrett G, Copas A, *et al.* London Measure of Unplanned Pregnancy: guidance for its use as an outcome measure. *Patient Relat Outcome Meas* 2017;**Volume 8**:43–56. doi:10.2147/prom.s122420

25 Vyas S, Kumaranayake L. Constructing socio-economic status indices: How to use principal components analysis. *Health Policy Plan* 2006;**21**:459–68. doi:10.1093/heapol/czl029

26 Manguro GO, Gichuki C, Ampt FH, *et al.* HIV infections among female sex workers in Mombasa, Kenya: current prevalence and trends over 25 years. *Int J STD AIDS* 2020;**31**:1389–97. doi:10.1177/0956462420950571

27 Ampt FH, Lim MSC, Agius PA, *et al.* Effect of a mobile phone intervention for female sex workers on unintended pregnancy in Kenya (WHISPER or SHOUT): a cluster-randomised controlled trial. *Lancet Glob Heal* 2020;**8**:e1534–45. doi:10.1016/S2214-109X(20)30389-2

28 Ampt FH, Lim MSC, Agius PA, *et al.* Use of long-acting reversible contraception in a cluster-random sample of female sex workers in Kenya. *Int J Gynecol Obstet* 2019;**146**:184–91. doi:10.1002/ijgo.12862

29 Ampt FH, Willenberg L, Agius PA, *et al.* Incidence of unintended pregnancy among female sex workers in low-income and middle-income countries: a systematic review and meta-analysis. *BMJ Open* 2018;**8**:21779. doi:10.1136/bmjopen-2018-021779

30 Makenzius M, Faxelid E, Gemzell-danielsson K, *et al.* Contraceptive uptake in post abortion care — Secondary outcomes from a randomised controlled trial , Kisumu , Kenya. 2018;**354**:1–13.

31 Swain SN, Saggurti N, Battala M, *et al.* Experience of violence and adverse reproductive health outcomes, HIV risks among mobile female sex workers in India. *BMC Public Health* 2011;**11**:357. doi:10.1186/1471-2458-11-357

32 McDougal L, Strathdee SA, Rangel G, *et al.* Adverse Pregnancy Outcomes and Sexual Violence Among Female Sex Workers Who Inject Drugs on the United States–Mexico Border. *Violence Vict* 2013;**28**:496–512. doi:10.1891/0886-6708.11-00129

33 Campbell MK, Piaggio G, Elbourne DR, *et al.* Consort 2010 statement: Extension to cluster randomised trials. *BMJ* 2012;**345**:19–23. doi:10.1136/bmj.e5661

34 Cheuk E, Isac S, Musyoki H, *et al.* Informing HIV prevention programs for adolescent girls and young women: A modified approach to programmatic mapping and key population size estimation. *J Med Internet Res* 2019;**21**:1–11. doi:10.2196/11196

For peer review only

Figure 1: Eligibility flow diagram for the WHISPER or SHOUT study as per Consort 2010 statement: extension to cluster randomized trials.[33]

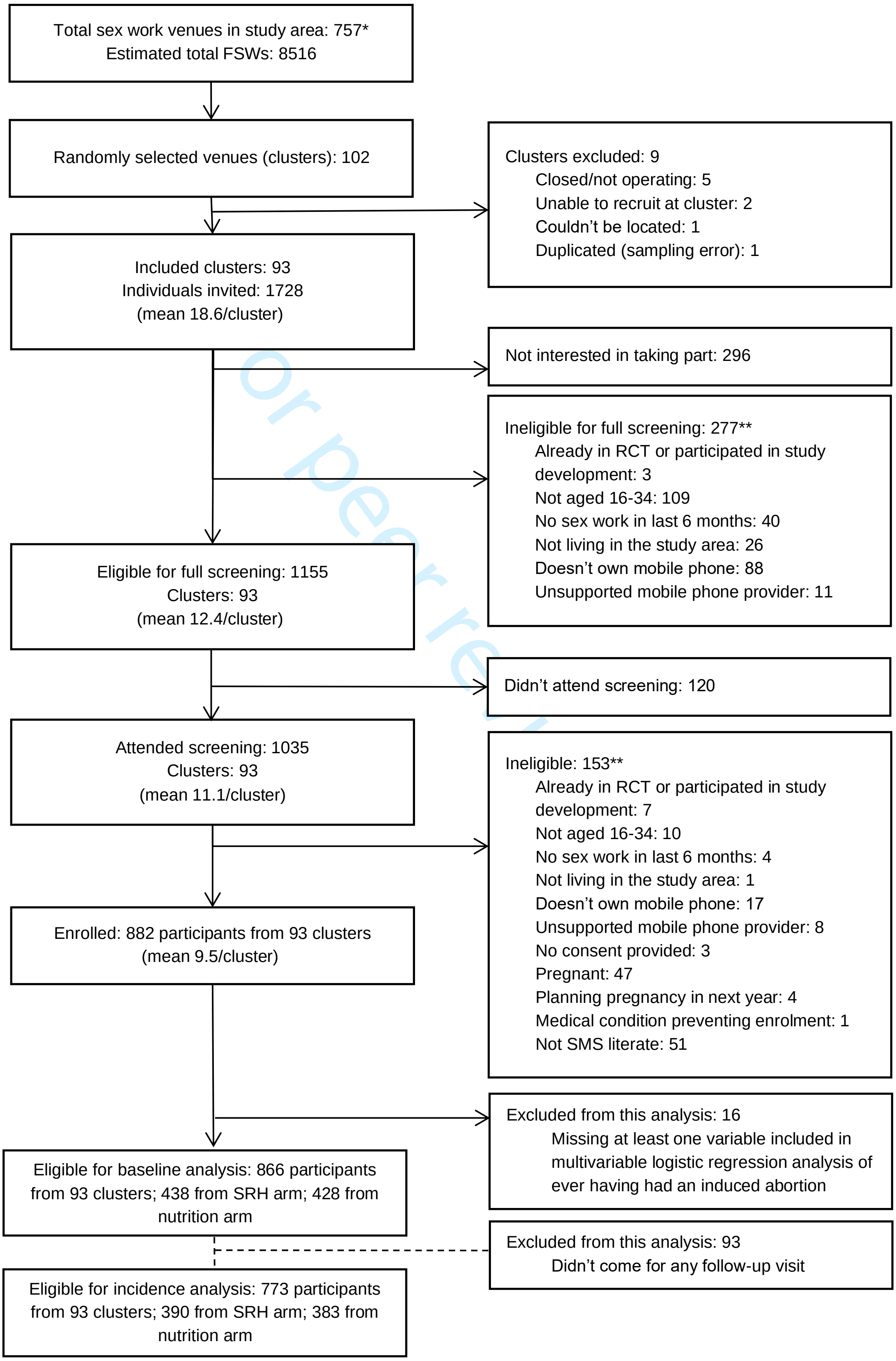

*Total hotspots and number per hotspot in study area enumerated by Cheuk et al.[34]
**One reason for ineligibility reported per participant and criteria determined in the order shown.

Figure 2: Overview of pregnancy outcomes during the 12-month follow-up. N=773

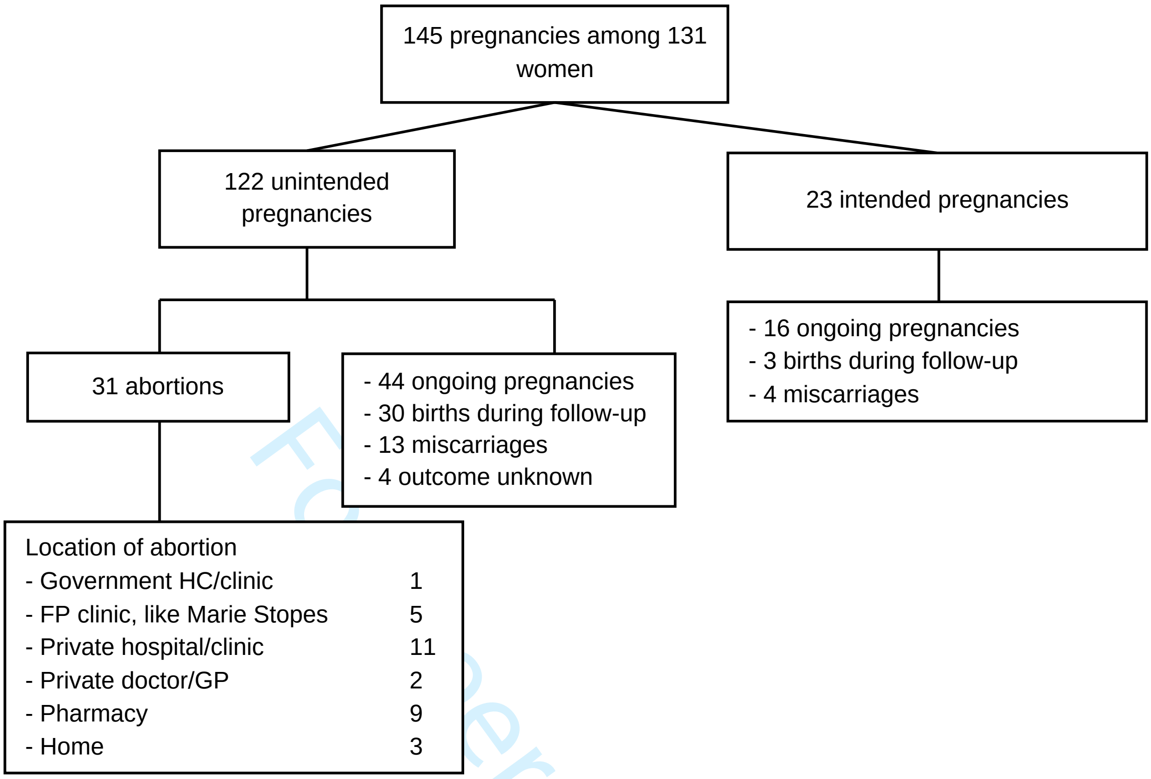

For peer review only

CONSORT 2010 checklist of information to include when reporting a cluster randomised trial:

Protocol paper[1] and primary outcome paper[2]:

1 Ampt FH, Mudogo C, Gichangi P, *et al.* WHISPER or SHOUT study: Protocol of a cluster-randomised controlled trial assessing mHealth sexual reproductive health and nutrition interventions among female sex workers in Mombasa, Kenya. *BMJ Open* 2017;**7**. doi:10.1136/bmjopen-2017-017388
2 Ampt FH, Lim MSC, Agius PA, *et al.* Effect of a mobile phone intervention for female sex workers on unintended pregnancy in Kenya (WHISPER or SHOUT): a cluster-randomised controlled trial. *Lancet Glob Heal* 2020;**8**:e1534–45. doi:10.1016/S2214-109X(20)30389-2

Section/Topic	Item No	Standard Checklist item	Extension for cluster designs	Page No *
**Title and abstract**				
	1a	Identification as a randomised trial in the title	Identification as a cluster randomised trial in the title	**1**
	1b	Structured summary of trial design, methods, results, and conclusions (for specific guidance see CONSORT for abstracts)[1,2]	See table 2	**2**
**Introduction**				
**Background and objectives**	2a	Scientific background and explanation of rationale	Rationale for using a cluster design	**5 (and reference to protocol paper**[1]**)**
	2b	Specific objectives or hypotheses	Whether objectives pertain to the the cluster level, the individual participant level or both	**4 (and reference to protocol paper**[1]**)**
**Methods**				
**Trial design**	3a	Description of trial design (such as parallel, factorial) including allocation ratio	Definition of cluster and description of how the design features apply to the clusters	**5 (and reference to protocol paper**[1]**)**
	3b	Important changes to methods after trial commencement (such as eligibility criteria), with reasons		**N/A**

**Participants**	4a	Eligibility criteria for participants	Eligibility criteria for clusters	**5 (and reference to protocol paper**[1]**)**
	4b	Settings and locations where the data were collected		**5**
**Interventions**	5	The interventions for each group with sufficient details to allow replication, including how and when they were actually administered	Whether interventions pertain to the cluster level, the individual participant level or both	**5 (and reference to protocol paper**[1]**)**
**Outcomes**	6a	Completely defined pre-specified primary and secondary outcome measures, including how and when they were assessed	Whether outcome measures pertain to the cluster level, the individual participant level or both	**4, 6 (and reference to protocol paper**[1]**)**
	6b	Any changes to trial outcomes after the trial commenced, with reasons		**N/A**
**Sample size**	7a	How sample size was determined	Method of calculation, number of clusters(s) (and whether equal or unequal cluster sizes are assumed), cluster size, a coefficient of intracluster correlation (ICC or *k*), and an indication of its uncertainty	**Reference to protocol paper**[1]
	7b	When applicable, explanation of any interim analyses and stopping guidelines		**N/A**
**Randomisation:**				
**Sequence generation**	8a	Method used to generate the random allocation sequence		**5 (and reference to protocol paper**[1]**)**
	8b	Type of randomisation; details of any restriction (such as blocking and block size)	Details of stratification or matching if used	**Reference to protocol paper**[1]

For peer review only

**Allocation concealment mechanism**	9	Mechanism used to implement the random allocation sequence (such as sequentially numbered containers), describing any steps taken to conceal the sequence until interventions were assigned	Specification that allocation was based on clusters rather than individuals and whether allocation concealment (if any) was at the cluster level, the individual participant level or both	**Reference to protocol paper**[1]
**Implementation**	10	Who generated the random allocation sequence, who enrolled participants, and who assigned participants to interventions	Replace by 10a, 10b and 10c	**5 (and reference to protocol paper**[1]**)**
	10a		Who generated the random allocation sequence, who enrolled clusters, and who assigned clusters to interventions	**5 (and reference to protocol paper**[1]**)**
	10b		Mechanism by which individual participants were included in clusters for the purposes of the trial (such as complete enumeration, random sampling)	**5**
	10c		From whom consent was sought (representatives of the cluster, or individual cluster members, or both), and whether consent was sought before or after randomisation	**5**
**Blinding**	11a	If done, who was blinded after assignment to interventions (for example, participants, care providers, those assessing outcomes) and how		**Reference to protocol paper**[1]
	11b	If relevant, description of the similarity of interventions		**5 (and reference to protocol paper**[1]**)**
**Statistical methods**	12a	Statistical methods used to compare groups for primary and secondary outcomes	How clustering was taken into account	**6**

For peer review only

	12b	Methods for additional analyses, such as subgroup analyses and adjusted analyses		**6**
**Results**				
**Participant flow (a diagram is strongly recommended)**	13a	For each group, the numbers of participants who were randomly assigned, received intended treatment, and were analysed for the primary outcome	For each group, the numbers of clusters that were randomly assigned, received intended treatment, and were analysed for the primary outcome	**Figure 1**
	13b	For each group, losses and exclusions after randomisation, together with reasons	For each group, losses and exclusions for both clusters and individual cluster members	**Figure 1**
**Recruitment**	14a	Dates defining the periods of recruitment and follow-up		**8**
	14b	Why the trial ended or was stopped		**5, 8**
**Baseline data**	15	A table showing baseline demographic and clinical characteristics for each group	Baseline characteristics for the individual and cluster levels as applicable for each group	**8, 9**
**Numbers analysed**	16	For each group, number of participants (denominator) included in each analysis and whether the analysis was by original assigned groups	For each group, number of clusters included in each analysis	**Figure 1, Table 1, 2 and 3**
**Outcomes and estimation**	17a	For each primary and secondary outcome, results for each group, and the estimated effect size and its precision (such as 95% confidence interval)	Results at the individual or cluster level as applicable and a coefficient of intracluster correlation (ICC or k) for each primary outcome	**Table 2 and 3**
	17b	For binary outcomes, presentation of both absolute and relative effect sizes is recommended		**Table 2 and 3**
**Ancillary analyses**	18	Results of any other analyses performed,		**Table 2 and 3**

For peer review only

		including subgroup analyses and adjusted analyses, distinguishing pre-specified from exploratory		
**Harms**	19	All important harms or unintended effects in each group (for specific guidance see CONSORT for harms[3])		**Reference to primary outcome paper**[2]
**Discussion**				
**Limitations**	20	Trial limitations, addressing sources of potential bias, imprecision, and, if relevant, multiplicity of analyses		**13, 14**
**Generalisability**	21	Generalisability (external validity, applicability) of the trial findings	Generalisability to clusters and/or individual participants (as relevant)	**12, 13**
**Interpretation**	22	Interpretation consistent with results, balancing benefits and harms, and considering other relevant evidence		**12, 13**
**Other information**				
**Registration**	23	Registration number and name of trial registry		**15**
**Protocol**	24	Where the full trial protocol can be accessed, if available		**Reference to protocol paper**[1] **(page 5)**
**Funding**	25	Sources of funding and other support (such as supply of drugs), role of funders		**2**

** Note: page numbers optional depending on journal requirements*

For peer review only

Table 2: Extension of CONSORT for abstracts[1,2] to reports of cluster randomised trials

Item	Standard Checklist item	Extension for cluster trials
**Title**	Identification of study as randomised	**Identification of study as cluster randomised**
**Trial design**	Description of the trial design (e.g. parallel, cluster, non-inferiority)	
**Methods**		
**Participants**	Eligibility criteria for participants and the settings where the data were collected	**Eligibility criteria for clusters**
**Interventions**	Interventions intended for each group	
**Objective**	Specific objective or hypothesis	**Whether objective or hypothesis pertains to the cluster level, the individual participant level or both**
**Outcome**	Clearly defined primary outcome for this report	**Whether the primary outcome pertains to the cluster level, the individual participant level or both**
**Randomization**	How participants were allocated to interventions	**How clusters were allocated to interventions**
**Blinding (masking)**	Whether or not participants, care givers, and those assessing the outcomes were blinded to group assignment	
**Results**		
**Numbers randomized**	Number of participants randomized to each group	**Number of clusters randomized to each group**
**Recruitment**	Trial status[1]	
**Numbers analysed**	Number of participants analysed in each group	**Number of clusters analysed in each group**
**Outcome**	For the primary outcome, a result for each group and the estimated effect size and its precision	**Results at the cluster or individual participant level as applicable for each primary outcome**
**Harms**	Important adverse events or side effects	
**Conclusions**	General interpretation of the results	
**Trial registration**	Registration number and name of trial register	
**Funding**	Source of funding	

[1] Relevant to Conference Abstracts

For peer review only

REFERENCES

[1] Hopewell S, Clarke M, Moher D, Wager E, Middleton P, Altman DG, et al. CONSORT for reporting randomised trials in journal and conference abstracts. *Lancet* 2008, 371:281-283

[2] Hopewell S, Clarke M, Moher D, Wager E, Middleton P, Altman DG at al (2008) CONSORT for reporting randomized controlled trials in journal and conference abstracts: explanation and elaboration. *PLoS Med* 5(1): e20

[3] Ioannidis JP, Evans SJ, Gotzsche PC, O'Neill RT, Altman DG, Schulz K, Moher D. Better reporting of harms in randomized trials: an extension of the CONSORT statement. *Ann Intern Med* 2004; 141(10):781-788.

For peer review only

BMJ Open

ASSESSMENT OF THE LIFETIME PREVALENCE AND INCIDENCE OF INDUCED ABORTION AND CORRELATES AMONG FEMALE SEX WORKERS IN MOMBASA, KENYA: A SECONDARY COHORT ANALYSIS

Journal:	*BMJ Open*
Manuscript ID	bmjopen-2021-053218.R2
Article Type:	Original research
Date Submitted by the Author:	09-Feb-2022

Complete List of Authors:	Simmelink, Anne Marieke; The Aga Khan University Hospital Nairobi, Department of Population Health<br>Gichuki, Caroline M.; The Aga Khan University Hospital Nairobi, Department of Population Health; International Centre for Reproductive Health Kenya<br>Ampt, Frances H.; Burnet Institute, Department of Epidemiology and Preventive Medicine; Monash University, Department of Epidemiology and Preventive Medicine<br>Manguro, Griffins; International Centre for Reproductive Health Kenya<br>Lim, Megan; Burnet Institute; Monash University, Department of Epidemiology and Preventive Medicine<br>Agius, Paul; Burnet Institute, Department of Epidemiology and Preventive Medicine; Monash University, Department of Epidemiology and Preventive Medicine<br>Hellard, Margaret; Burnet Institute, Department of Epidemiology and Preventive Medicine; Monash University, Department of Epidemiology and Preventive Medicine<br>Jaoko, Walter; University of Nairobi, Department of Medical Microbiology<br>Stoové, Mark; Burnet Institute, Department of Epidemiology and Preventive Medicine; Monash University, Department of Epidemiology and Preventive Medicine<br>L'Engle, Kelly; University of San Francisco<br>Temmerman, Marleen; International Centre for Reproductive Health Kenya; The Aga Khan University Hospital Nairobi, Department of Obstetrics and Gynaecology<br>Gichangi, Peter; International Centre for Reproductive Health Kenya; Ghent University, Department of Public Health and Primary Care<br>Luchters, Stanley; The Aga Khan University Hospital Nairobi, Department of Population Health; Burnet Institute, Department of Population Health

**Primary Subject Heading**:	Sexual health
Secondary Subject Heading:	Sexual health, Public health
Keywords:	SEXUAL MEDICINE, PUBLIC HEALTH, EPIDEMIOLOGY

SCHOLARONE™
Manuscripts

I, the Submitting Author has the right to grant and does grant on behalf of all authors of the Work (as defined in the below author licence), an exclusive licence and/or a non-exclusive licence for contributions from authors who are: i) UK Crown employees; ii) where BMJ has agreed a CC-BY licence shall apply, and/or iii) in accordance with the terms applicable for US Federal Government officers or employees acting as part of their official duties; on a worldwide, perpetual, irrevocable, royalty-free basis to BMJ Publishing Group Ltd ("BMJ") its licensees and where the relevant Journal is co-owned by BMJ to the co-owners of the Journal, to publish the Work in this journal and any other BMJ products and to exploit all rights, as set out in our licence.

The Submitting Author accepts and understands that any supply made under these terms is made by BMJ to the Submitting Author unless you are acting as an employee on behalf of your employer or a postgraduate student of an affiliated institution which is paying any applicable article publishing charge ("APC") for Open Access articles. Where the Submitting Author wishes to make the Work available on an Open Access basis (and intends to pay the relevant APC), the terms of reuse of such Open Access shall be governed by a Creative Commons licence – details of these licences and which Creative Commons licence will apply to this Work are set out in our licence referred to above.

Other than as permitted in any relevant BMJ Author's Self Archiving Policies, I confirm this Work has not been accepted for publication elsewhere, is not being considered for publication elsewhere and does not duplicate material already published. I confirm all authors consent to publication of this Work and authorise the granting of this licence.

For peer review only

ASSESSMENT OF THE LIFETIME PREVALENCE AND INCIDENCE OF INDUCED ABORTION AND CORRELATES AMONG FEMALE SEX WORKERS IN MOMBASA, KENYA: A SECONDARY COHORT ANALYSIS

A. Marieke Simmelink[1], Caroline M. Gichuki[1,2], Frances H. Ampt[3,4], Griffins Manguro[2], Megan S.C. Lim[3,4], Paul A. Agius[3,4], Margaret Hellard[3,4,5,6], Walter Jaoko[7], Mark Stoové[3,4,8], Kelly L'Engle[9], Marleen Temmerman[2,10,11], Peter Gichangi[2,10,12], Stanley Luchters[1,3,4,10]

AFFILIATIONS

1. Department of Population Health, Medical College, Aga Khan University, Nairobi, Kenya
2. International Centre for Reproductive Health (ICRH), Mombasa, Kenya
3. Burnet Institute, Melbourne, Australia
4. Department of Epidemiology and Preventive Medicine, Monash University, Melbourne, Australia
5. Department of Infectious Diseases, The Alfred Hospital, Melbourne, Australia
6. Doherty Institute and School of Population and Global Health, University of Melbourne, Australia
7. Department of Medical Microbiology, University of Nairobi, Nairobi, Kenya
8. School of Psychology and Public Health, La Trobe University, Melbourne, Australia
9. University of San Francisco, San Francisco, California, USA
10. Department of Public Health and Primary Care, Ghent University, Ghent, Belgium
11. Department of Obstetrics and Gynaecology, Aga Khan University, Kenya
12. Technical University of Mombasa, Mombasa, Kenya

CORRESPONDING AUTHOR

Stanley Luchters, Chair of the Department of Population Health, Medical College, Aga Khan University, Nairobi, Kenya. Email: stanley.luchters@aku.edu

Word count: 3037

For peer review only

For peer review only - http://bmjopen.bmj.com/site/about/guidelines.xhtml

STRUCTURED ABSTRACT

Introduction

Prevalence of lifetime induced abortion in female sex workers (FSWs) in Kenya was previously estimated between 43 and 86%. Our analysis aimed at assessing lifetime prevalence and correlates, and incidence and predictors of induced abortions among FSWs in Kenya.

Methods

This is a secondary prospective cohort analysis using data collected as part of the WHISPER or SHOUT cluster-randomized trial in Mombasa (Australian New Zealand Clinical Trials Registry number: ACTRN12616000852459), assessing effectiveness of an SMS-intervention to reduce incidence of unintended pregnancy. Eligible participants were current FSWs, 16-34 years and not pregnant or planning pregnancy. Baseline data on self-reported lifetime abortion, correlates and predictors were collected between September 2016 and May 2017. Abortion incidence was measured at six- and twelve-months follow-up. A multivariable logistic regression model was used to assess correlates of lifetime abortion and discrete-time survival analysis was used to assess predictors of abortions during follow-up.

Results

Among 866 eligible participants, lifetime abortion prevalence was 11.9%, while lifetime unintended pregnancy prevalence was 51.2%. Correlates of lifetime abortions were currently not using a highly effective contraceptive (AOR=1.76 [95%CI=1.11-2.79] p=0.017) and having ever experienced intimate partner violence (IPV) (AOR=2.61 [95%CI=1.35-5.06] p=0.005). Incidence of unintended pregnancy and induced abortion were 15.5 and 3.9 per 100 women-years, respectively. No statistically significant associations were found between hazard of abortion and age, sex work duration, partner status, contraceptive use and IPV experience.

Conclusion

Although experience of unintended pregnancy remains high, lifetime prevalence of abortion may have decreased among FSW in Kenya. Addressing IPV could further decrease induced abortions in this population.

KEYWORDS

Sex Work, induced abortion, unintended pregnancy, contraception, cluster-random sample, Kenya

FUNDING STATEMENT

For peer review only - http://bmjopen.bmj.com/site/about/guidelines.xhtml

66 This work was supported by Australia's National Health and Medical Research Council
67 (NHMRC), Project Grant GNT 1087006.

For peer review only

ARTICLE SUMMARY

Strengths and limitations

- This study presents incidence of abortion in a cluster-randomised cohort of FSWs.
- It is the first to analyse predictors of abortions in FSWs, rather than correlates of past abortions only.
- This paper explores a secondary research question, and the study was not originally powered to assess the predictors of abortions during follow-up.
- The sensitive topic of abortions and SRH in general, might have resulted in a social desirability bias.

For peer review only

For peer review only - http://bmjopen.bmj.com/site/about/guidelines.xhtml

INTRODUCTION

Research findings show that about 5% of Kenya's urban female reproductive population could be involved in sex work.[1] Female sex workers (FSWs) experience higher than average rates of HIV, other sexually transmitted infections (STIs) and unintended pregnancy.[2–5]

Unintended pregnancies often have negative consequences for FSWs, including financial adversity, social stigma and induced abortion.[4] In countries where abortion is illegal or difficult to access, women frequently resort to unsafe practices, risking severe medical complications.[6]

Reported prevalence of lifetime abortion among FSWs in low- and middle-income countries (LMICs) varies from 24% in Laos in 2012 to 86% in 2000/01 in Central and Western Kenya.[7–16] Many of these abortions are unsafe or sought in the informal sector.[8,11,16] In Mombasa, lifetime abortion among FSWs was estimated at 43% in 2008.[17]

In 2010, Kenya liberalized its abortion law, making abortion legal when "there is need for emergency treatment, or the life or health of the mother is in danger".[18] In practice, older laws criminalizing abortion remain in place, creating ambiguity among health professionals with lawsuits remaining a threat.[19] Moreover, social, cultural and religious beliefs condemning pregnancy termination, misconceptions about the illegality and costs of the procedure, still hamper access to safe services for women.[4,19]

A national study in 2012 estimated an induced abortion rate of 48 per 1,000 women among women aged 15-49, based on data of women who sought care for abortion complications.[20] It showed a diverse sociodemographic and economic background among these women in terms of educational level, employment status, marital status and religion.

Incidence of induced abortions among FSWs has not been studied in Kenya and studies analysing correlates of induced abortions elsewhere have been cross-sectional and report correlates of lifetime abortions, precluding attribution of causality.[8–15,21] Examining incidence of abortions and identifying predictors will help inform future policies to improve care around abortions for FSWs and the need for longitudinal data about abortions in FSWs has therefore been recognised.[8,14]

This secondary data analysis aimed to examine lifetime prevalence and correlates, and incidence and predictors of induced abortions in a cluster-random sample of FSWs in Mombasa, Kenya.

For peer review only - http://bmjopen.bmj.com/site/about/guidelines.xhtml

MATERIAL AND METHODS

Study design

This is a secondary prospective cohort analysis using data collected as part of the WHISPER or SHOUT cluster-randomized trial in Mombasa. A detailed description of the study protocol can be found elsewhere.[22] In summary, the study was a two-arm cluster-randomized controlled trial assessing the effectiveness of two SMS-based interventions targeting sexual and reproductive health (SRH) and nutrition in FSWs in Mombasa, Kenya. The study was conducted in two sub-counties in Mombasa, Kisauni and Changamwe, between September 2016 and July 2018. Ninety-three venues were randomly sampled with a probability proportional to FSW population size at the venue (Figure 1). Trained community mobilisers and peer educators recruited FSWs from the venues until the required sample size of 860 FSWs was achieved. This study uses baseline and follow-up data from the trial. During the recruitment period of 14 September 2016 to 16 May 2017, 882 women were enrolled in the study. Follow-up continued until 31 July 2018. A sub-sample of 866 women (98.2%) was analysed for this secondary analysis (Figure 1).

Study participants and procedures

Women were eligible for the study if they were aged 16-34 years; self-reported to have engaged in paid sex work in the last 6 months; were reportedly not pregnant or planning pregnancy within the next 12 months; resided within the study area; and were able to read text messages in basic English. Study-specific community mobilizers visited the selected clusters to recruit FSWs and conducted pre-screening interviews identifying women who self-reported to be sex workers. Potentially eligible FSWs were referred to the nearest study clinic for a clinical assessment, including a urine pregnancy test, and STI and HIV testing. Enrolled participants then completed a structured questionnaire administered in Swahili by trained research assistants, who had previously participated in research with FSWs. The questionnaire captured detailed sociodemographic, sexual and reproductive health information. Follow-up visits were scheduled at 6 and 12 months after enrolment. Procedures at follow-up visits were similar to those done at enrolment.

Participants received two to three SMS messages per week for 12 months. The messages consisted of stand-alone push messages, role model stories and on-demand messages, accessed using assigned codes. Participants only received and accessed messages on their phones from their assigned intervention.

Outcomes and correlates

Lifetime prevalence of induced abortion was assessed at baseline with the question 'How many times have you ever had an induced abortion?'. Induced abortions were assessed

For peer review only

during follow-up, by asking participants if they had been pregnant since their last visit. The outcome of each reported pregnancy was then assessed, and in the case of an induced abortion, the location was documented. Formal sector abortions were those taking place at a government or private hospital/clinic, a private doctor/General Practitioner (GP) or a Family Planning (FP) clinic. Informal sector abortions were defined as those taking place at home, a pharmacy or traditional healer.[23]

Pregnancies during follow-up were confirmed with a urine pregnancy test at the study clinic, or self-reported by the participant when occurring between study visits. Pregnancy intention for all reported pregnancies was assessed using the London Measure of Unintended Pregnancy (LMUP), a six-item scale. A pregnancy scoring less than 10 out of 12 on the LMUP was defined as unintended.[24]

All correlates of lifetime induced abortions and predictors of incident induced abortions were self-reported at baseline. Use of a highly effective contraceptive method was defined as use of contraceptive implants, IUD, injection, oral contraceptive pill and sterilization. High knowledge on FP was defined as answering five out of six true-false statements on FP correctly. Having a positive attitude on FP was defined as agreeing with at least three out of four positive attitude statements. Self-efficacy in FP was defined as high when agreeing with two statements on this topic. Household socio-economic status tertiles were generated using principal component analysis, based on 12 household assets.[25] Prevalence of intimate partner violence (IPV) was assessed by asking if the participant had ever been pushed, slapped, hit or kicked by a partner, or had been physically forced to have sex, agreed to sex out of fear of the consequences or forced to do something sexual that she perceived as degrading or humiliating. For each item, it was also assessed if this happened in the previous 12 months.

Statistical analysis

Statistical analysis was performed using Stata software version 14 (StataCorp, College Station, TX, USA). Distributions of socio-demographic characteristics, reproductive history, contraceptive use and intimate partner violence at baseline were explored with means and standard deviations for continuous variables and proportions for categorical variables. Covariates age, education level, SES-tertile, duration of sex work, having a husband/boyfriend, use of highly effective contraceptives and experience of IPV, were included in the multivariate models on the basis of a review of literature.[8–15,21] Covariates high FP knowledge, positive attitude to FP and high FP specific self-efficacy were included in the multivariate models on the basis of theoretical assumptions by the co-authors. It was hypothesised that these characteristics would be positively associated with contraceptive use and would protect against experiencing an induced abortion. Correlates of lifetime

For peer review only - http://bmjopen.bmj.com/site/about/guidelines.xhtml

abortion were identified using weighted multivariable logistic regression. Associations were considered statistically significant at the 5% level. The outcome incident abortion was interval-censored (measured at 6-monthly intervals). Therefore, a discrete-time survival analysis was performed using generalized linear mixed (GLM) modelling with complementary log-log link function and binomial distribution, a method that produces estimated hazard ratios. Abortions during follow-up were analysed for all participants who attended at least one follow-up visit. All outcomes presented here are cluster-adjusted, based on inverse probability sample means to account for sampling bias.

Ethical consideration

All study participants provided written informed consent. The study was approved by the Kenyatta National Hospital and University of Nairobi Ethics and Research Committee, Kenya (KNH-UoN ERC—KNH-ERC/RR/493) and the Monash University Human Research Ethics Committee, Australia (MUHREC—CF16/1552—2016000812).

Patient and public involvement

Patients or the public were not directly involved in the design, or conduct, or reporting, or dissemination plans of our research.

For peer review only

RESULTS

Mean age was 25.5 years (SD=4.7) (Table 1). The majority of women (n=765, 88.7%) had at least completed primary education and 306 (34.9%) had completed secondary education. Just over half of the participants (n=484, 56.6%) reported to have a current husband or boyfriend, but 812 (94.0%) reported not to live with a partner. Mean duration of employment in sex work was 4.7 years (SD=3.5). Among women currently reporting a husband or boyfriend, 344/483 (70.8%) had not disclosed their employment in sex work to their partners. 605/861 women (68.9%) worked fulltime in sex work and 508/863 (59.8%) earned more than 2000 Ksh (about USD $20) per week from sex work. The majority of women (n=666, 76.1%) had ever been pregnant, and 451 (51.2%) ever had an unintended pregnancy. 103/866 (11.9%) reported to have had at least one induced abortion in their lifetime. Among women who had an induced abortion, 58/102 (57.1%) went to a private hospital or clinic for the most recent abortion. 29/102 (29.1%) women had their most recent abortion in the informal sector, like home, a pharmacy or traditional healer. At baseline, 463 women (54.4%) reported to use a highly effective contraceptive method. Three-quarters of FSW (650/866; 75.0%) ever experienced IPV and 525/866 (60.1%) experienced IPV in the past 12 months the before the baseline questionnaire was conducted.

Table 1. Socio-demographic characteristics, reproductive history, contraceptive use and intimate partner violence at baseline of female sex workers in Mombasa, Kenya (n=866, unless stated otherwise)

Characteristic	n	Cluster-adjusted mean (SD) or proportion in % (95% CI)[a]
Mean age, in years		25.5 (4.7)
Highest level of education		
None or some primary	101	11.2 (9.2-13.6)
Completed primary or some secondary	459	53.8 (50.1-57.6)
Completed secondary or some tertiary	306	34.9 (31.3-38.7)
Religion (n=864)		
Protestant	389	44.8 (41.4-48.2)
Catholic	304	36.0 (32.3-39.8)
Muslim	171	19.2 (15.6-23.4)
Electricity available in household (n=863)[b]	660	76.4 (73.0-79.5)
Duration of sex work, in years		4.7 (3.5)
Fulltime FSW (n=861)[c]	605	68.9 (64.0-73.5)
Weekly income from sex work (n=861)		
≤1000 Ksh	144	16.2 (13.2-19.8)
1001-2000 Ksh	211	24.0 (21.0-27.3)

For peer review only - http://bmjopen.bmj.com/site/about/guidelines.xhtml

≥2001 Ksh[d]	508	59.8 (55.1-64.3)
Sex work venue		
Bar with lodging	388	43.8 (37.7-50.2)
Bar without lodging	147	17.2 (13.7-21.3)
Lodging/guesthouse	138	15.1 (10.9-20.5)
Street/beach	86	11.2 (7.8-15.7)
Other[e]	107	12.7 (9.1-17.5)
Marital status		
Married/cohabiting	54	6.0 (4.5-8.0)
Single (not cohabiting)	627	73.0 (69.6-76.2)
Separated/divorced/widowed	185	21.0 (18.0-24.3)
Currently has husband/boyfriend	484	56.6 (52.5-60.7)
Disclosure of sex work to husband/boyfriend (n=483)[f]		
Yes	136	28.7 (24.4-33.3)
No	344	70.8 (66.0-75.1)
Don't know	3	0.6 (0.2-1.7)
Ever had a pregnancy	666	76.1 (72.3-79.5)
Has a living child	622	71.2 (67.1-75.0)
Ever had an unintended pregnancy (N=864)[g]	451	51.2 (47.4-54.9)
Ever had an induced abortion	103	11.9 (10.0-14.2)
Location of most recent induced abortion (N=102)		
Government hospital	2	1.8 (0.4-6.9)
FP clinic, like Marie Stopes	9	8.3 (4.4-15.0)
Private hospital/clinic	58	57.1 (46.5-67.2)
Private doctor GP	4	3.7 (1.4-9.6)
Pharmacy	9	8.9 (4.6-16.8)
Traditional healer	4	3.8 (1.4-10.0)
Home	16	16.4 (9.0-27.8)
Uses a highly effective contraceptive method[h]	473	54.4 (49.5-59.2)
Ever experienced IPV[i]	650	75.0 (71.1-78.5)
Experienced IPV in past 12 months[j]	525	60.1 (55.5-64.6)

[a] Inverse probability-weighted percentages. [b] Availability of electricity in the household is presented here as a proxy for household SES. [c] Fulltime work as FSW is characterized as having no other sources of income in the last 6 months. [d] 1000 Kenyan Shilling (Ksh) is about USD $10. [e] Brothel, strip club, casino, massage parlors, parks or home. [f] Among participants with a husband or boyfriend. [g] Assessed using the London Measure of Unintended Pregnancy (LMUP) [h] Highly effective is defined as use of contraceptive implants, IUD, injection, oral contraceptive pill and sterilization. [i] IPV= intimate partner violence. [j] Before baseline.

Women currently not using a highly effective contraceptive (AOR=1.76 [95%CI=1.11-2.79] p=0.017) and women who ever experienced IPV (AOR=2.61 [95%CI=1.35-5.06] p=0.005) were significantly more likely to report a history of induced abortion, when controlled for potential confounders (Table 2). Longer duration of sex work showed a borderline positive association with history of abortion (AOR=1.08 [95%CI=1.00-1.16] p=0.053). Although

For peer review only

For peer review only - http://bmjopen.bmj.com/site/about/guidelines.xhtml

higher age was significantly associated with a history of abortion in bivariate analysis, after adjusting for confounding factors this association was no longer seen.

Table 2. Correlates of participants with a history of induced abortion, and cluster-adjusted bi- and multivariable logistic regression analysis on history of induced abortion (n=866)

Characteristic		Crude Odds Ratio (OR)		Adjusted OR	
	Ever had an induced abortion (n=103); n/N (cluster-adjusted proportion in %)[a]	OR (95% CI)[b]	p-value	OR (95% CI)[b]	p-value
Age (in years)	27.0 (4.9)[c]	1.08 (1.03-1.14)	**0.001**	1.04 (0.97-1.11)	0.280
Highest level of education					
None or some primary	14/101 (13.5)	Ref.		Ref.	
Completed primary or some secondary	57/459 (12.4)	0.90 (0.47-1.72)	0.749	0.96 (0.48-1.99)	0.895
Completed secondary or some tertiary	32/306 (10.7)	0.77 (0.40-1.47)	0.423	0.83 (0.40-1.84)	0.620
SES-tertile[e]					
Poorest	39/290 (13.4)	Ref.		Ref.	
Middle	33/287 (10.9)	0.79 (0.46-1.35)	0.386	0.89 (0.50-1.59)	0.697
Richest	31/289 (11.4)	0.83 (0.47-1.46)	0.510	0.83 (0.46-1.50)	0.674
Mean duration of sex work (in years)	6.1 (3.4)[c]	1.12 (1.07-1.17)	**<0.001**	1.08 (1.00-1.16)	0.053
Highly effective contraceptive use[d]					
Yes	51/473 (10.6)	Ref.		Ref.	
No	52/393 (13.5)	1.32 (0.88-1.97)	0.173	1.76 (1.11-2.79)	**0.017**
High FP knowledge score					
No	60/562 (10.5)	Ref.		Ref.	
Yes	43/304 (14.5)	1.44 (0.95-2.19)	0.084	1.34 (0.85-2.10)	0.200
Positive attitude to FP use					
No	43/354 (12.4)	Ref.		Ref.	
Yes	60/512 (11.6)	0.93 (0.60-1.44)	0.743	0.90 (0.56-1.45)	0.661
High FP-specific self-efficacy					
No	24/237 (10.1)	Ref.		Ref.	
Yes	79/629 (12.6)	1.28 (0.76-2.17)	0.345	1.23 (0.72-2.10)	0.454
Ever experienced intimate partner violence					
No	12/216 (5.2)	Ref.		Ref.	
Yes	91/650 (14.1)	2.98 (1.55-5.74)	**0.001**	2.61 (1.35-5.06)	**0.005**

[a] Inverse probability-weighted percentages. [b] Standard errors are corrected by cluster sandwich variance estimation. [c] Mean (SD) of women who ever had an induced abortion. [d] Highly effective is defined as use of contraceptive implants, IUD, injection, oral contraceptive pill and sterilization. [e] SES = Socio-economic status.

During the study follow-up, 773 women attended at least one follow-up visit (Figure 1). Total follow-up time was 9,468 months, with an average of 12.2 months per woman (data not shown). A total of 131 participants became pregnant, with a total of 145 pregnancies among these women (Figure 2). Of these pregnancies, 122/145 were unintended according to the LMUP. Among 145 pregnancies, 31 ended in induced abortion, among 29 women and

For peer review only

across 789 women-years at risk. Overall incidence rate was 3.9 induced abortions per 100 women-years of observation. Out of 31 abortions, 19 took place in the formal sector and 12 in an informal setting.

The GLM modelling of abortion incidence showed that women experiencing IPV in the past year (HR=1.93 [95%CI=0.86-4.34] p=0.122) and women not using a highly effective contraceptive (HR=1.51 [95%CI=0.66-3.49] p=0.332) exhibited a higher hazard of abortion, independent of other factors, although these results were not significant (Table 3). We did not find a relation between age, mean duration of sex work, currently having a husband or boyfriend and the intervention under study and hazard of induced abortion.

Table 3. Baseline predictors of incident abortion in FSWs among Mombasa, Kenya (N=773)[a]

**Baseline predictors of incident abortions**	**Unadjusted[b]**		**Adjusted HR[c]**	
	**HR (95% CI)[d]**	**p-value**	**HR (95% CI)[d]**	**p-value**
Age (in years)	0.96 (0.89-1.04)	0.315	1.00 (0.91-1.09)	0.918
Mean duration of sex work (in years)	0.93 (0.82-1.05)	0.234	0.92 (0.79-1.09)	0.336
Currently has husband/boyfriend	0.83 (0.40-1.73)	0.622	0.80 (0.39-1.64)	0.537
Not using highly effective contraceptive[e]	1.50 (0.69-3.23)	0.310	1.51 (0.66-3.49)	0.332
Experienced IPV in last 12 months	1.67 (0.74-3.79)	0.216	1.93 (0.86-4.34)	0.122

[a] Discrete-time survival analysis including the first induced abortion per women. Generalized linear mixed model with complementary log-log link, binomial distribution, offset for log time between visits and random intercept for participants. [b] HR = Hazard Ratio. [c] All adjusted Hazard Ratios are also adjusted for the intervention. The intervention had no detectable effect on the outcome of incident abortions. [d] Cluster robust standard errors for sex-work venue clustering. [e] Highly effective is defined as use of contraceptive implants, IUD, injection, oral contraceptive pill and sterilization.

For peer review only

DISCUSSION

This study adds to the current knowledge of abortion practices in FSWs. Lifetime induced abortion prevalence in this population was 11.9%. This seems considerably lower than previous figures of lifetime abortion of 86% in 2004 in central and western Kenya among FSWs of a similar age, and 43% in 2008 in Mombasa among FSWs who were on average 2 years older.[7,17] In the former study, it was not specified if these abortions also included spontaneous abortions, which might have overestimated the prevalence of abortions. However, despite the sociodemographic and methodological differences between the studies, the size of the difference is suggestive of an actual lower rate in abortions in this population. The prevalence of abortions found here is also lower than reports from other LMICs ranging between 24% and 64% in Laos and Cote d'Ivoire, respectively.[8–16] A possible explanation for this lower prevalence is the relatively high use of highly effective contraceptives of 54% in our cohort, compared to similar studies from LMICs. [8,12,14–16] The findings are furthermore consistent with a lower-than-expected HIV prevalence and unintended pregnancy incidence in our cohort and could be a result of peer-mediated interventions implemented over the past years in the Mombasa area.[26,27] These have mostly targeted prevention of HIV and STIs, but likely have had a lowering effect on unintended pregnancies and induced abortions as well.[22,26] Furthermore, this study, in contrast to above referenced studies, attempted to draw a representative sample of a FSW population from community settings, whereas other studies used non-probability sampling methods, which might have overestimated past abortions.

Despite the lower-than-expected unintended pregnancy incidence, still 51% of FSWs in our cohort reported an unintended pregnancy in their lifetime. The gap between lifetime unintended pregnancies and lifetime induced abortions could indicate that many women decide to keep a child from unintended pregnancies, which could be supported by the fact that between 70-80% of young FSW in Mombasa have reported one or more children.[4,27] It may also indicate a high unmet need for induced abortion services among FSWs, for example due to ongoing or increasing difficulties in accessing SRH or abortion services for this group or increasing sociocultural barriers to abortion. Barriers to accessing other SRH services such as long-acting reversible contraceptives has previously been reported for this population.[28]

The present study is one of the few studies to report incidence of abortion among FSWs and to our knowledge, the first to analyse predictors of abortions in FSWs, rather than correlates of past abortions only. Incidence of induced abortion in our cohort was 3.9 per 100 women-years. Compared to other studies from LMICs, this is similar to two studies reporting abortion incidence rates of 3.1 and 3.0 per 100 women-years and lower than a third study reporting 7.4 induced abortions per 100 women-years among FSWs.[29] The intervention

For peer review only - http://bmjopen.bmj.com/site/about/guidelines.xhtml

under study had no measurable effect on unintended pregnancy incidence and is therefore unlikely to have affected incidence of induced abortions.[27]

Informal sector abortions where common in this cohort, with 29% of women having had their most recent abortion in the informal sector, and 39% of the reported abortions during follow-up happening in the informal sector. These informal sector abortions, put women at higher risk of complications due to unsafe practices and this denotes a need for information on safer alternatives, like the Marie Stopes clinics.[23]

Multiple studies have found both age and duration of sex work to be correlated to past abortions. Commonly higher age [8,11,15] and longer duration [9,12] of sex work were associated with higher lifetime abortion prevalence. One study found that younger age was associated with past abortions.[10] In our cohort, although FSWs with a past abortion in our cohort were older in the crude analysis, after adjusting for other correlates, this difference was no longer significant. The association with longer duration of sex work remained borderline significant in multivariate analysis. We did not find a relation between age and mean duration of sex work and having an induced abortion during follow-up. This might suggest that the association between past abortions and higher age and longer duration of sex work is caused by cumulative exposure to high risk of pregnancies and abortion.

We found a positive association between currently not using a highly effective contraceptive and having a past abortion. No difference was found in FP-specific self-efficacy or knowledge, or attitude towards FP among women with and without a past abortion. The found association could indicate significant barriers to uptake or continuation of a highly effective contraceptive method post abortion, as has previously been acknowledged by a study in Kenya.[30]

In our cohort, experience of IPV was high and the odds of having had a past abortion were more than 2.5-times as high for women who experienced IPV in the past, consistent with findings from other studies.[14,15] Our study also showed a positive association between experience of IPV in the past twelve months and abortions during follow-up, but this was not significant. Experience of (intimate partner) violence has been shown to have a negative effect on the reproductive health of FSWs, with greater risks of adverse pregnancy outcomes and forced termination of pregnancy.[31,32] Addressing the problem of IPV in this population could further lower induced abortions.

Limitations

Some limitations should be considered when interpreting the findings from this study. The sensitive topic of abortions and SRH in general, might have resulted in a social desirability bias. To minimize this, peer-educators and research assistants had previous experience working with the target population and received additional training. Attrition bias might have

For peer review only - http://bmjopen.bmj.com/site/about/guidelines.xhtml

occurred due to loss to follow-up of pregnant participants, as has been recognized by anecdotal evidence.[27] This might have resulted in an underestimation of abortions in our study. The robust multi-stage sampling method improved the ability to generalise the findings to the larger sex work population in the Coast region. However, this study was done in a well-researched population, targeted by other peer-mediated interventions in the past two decades, which may limit generalisation to sex worker populations in other settings. A further limitation is that this paper explores a secondary research question, and the study was not originally powered to assess the predictors of abortions during follow-up. Unknown timing of the past abortions in relation to studied correlates, restrict judgement of temporality of the studied associations. Lastly, measurements of abortions stopped when the intervention stopped, so the actual number of abortions during follow-up might in fact be higher than captured in the study.

Suggestions for further research

Future research is needed to explore the trend in abortion incidence among FSWs in Kenya. In order to improve care, we need to better understand current abortion practices, the decision-making process around terminating unintended pregnancies, how uptake of highly effective contraceptives can be increased post-abortion, as well as the relationship between experience of IPV and induced abortions.

CONCLUSIONS

In conclusion, the prevalence of lifetime induced abortions in a random cohort of FSWs in Mombasa, was 11.9% and incidence was 3.9 per 100 women-years, whereas prevalence and incidence of unintended pregnancies were higher at 51% and 15.5 per 100 women-years, respectively. A history of induced abortion was positively associated with not using a highly contraceptive method at baseline and having experienced IPV in the past. The study did not find a significant association with the studied predictors of abortions.

For peer review only - http://bmjopen.bmj.com/site/about/guidelines.xhtml

ACKNOWLEDGEMENTS

The authors would like to acknowledge the hard work of the field research team, specifically Christine Maghanga, Millicent Okello, Betty Kitili, Judith Wamaua, Elizabeth Bilasi, Marion Mwangi, Promillah Muindi, Rukia Abdallah and Khadija Kassim, as well as all the study participants of the study. The authors gratefully acknowledge the contribution of funding from Australia's National Health and Medical Research Council for the WHISPER or SHOUT trial (Project Grant GNT 1087006), Career Development Fellowships for SL, Senior Research Fellowship for MS, and a Postgraduate Scholarship for FHA. The sponsor did not contribute to study design; data collection, analysis, or interpretation; manuscript writing; or the decision to submit the article for publication. Lastly, we acknowledge the contribution of funding from the Victorian Operational Infrastructure Support Program received by the Burnet Institute.

TRIAL REGISTRATION

The trial was registered in the Australian New Zealand Clinical Trials Registry, ACTRN12616000852459.

AUTHOR CONTRIBUTORSHIP

SL was the Principal Investigator on the study. SL, FHA, PG, MSCL, PAA, MH, WJ, MS, KL, MT and MFC contributed to the study design. CG and GM coordinated the trial and undertook data acquisition in Kenya under the supervision of PG. AMS and SL conceptualized the manuscript. AMS and PAA conducted the statistical analyses. AMS and CG wrote the first draft of the manuscript. All authors contributed to data interpretation, provided critical input, and approved the final version of the manuscript.

DECLARATION OF INTERESTS

The authors have no conflicts of interest.

DATA AVAILABILITY STATEMENT

The original data are not available in a public repository. The corresponding author is to be contacted for the consideration of any data requests.

For peer review only

392 **FIGURE CAPTIONS**

393 **Figure 1:** Eligibility flow diagram for the WHISPER or SHOUT study as per Consort 2010
394 statement: extension to cluster randomized trials.[33,34]

395 **Figure 2:** Overview of pregnancy outcomes during the 12-month follow-up. N=773

For peer review only

For peer review only - http://bmjopen.bmj.com/site/about/guidelines.xhtml

REFERENCES

1 Odek WO, Githuka GN, Avery L, *et al.* Estimating the size of the female sex worker population in Kenya to inform HIV prevention programming. *PLoS One* 2014;**9**. doi:10.1371/journal.pone.0089180

2 Baral S, Beyrer C, Muessig K, *et al.* Burden of HIV among female sex workers in low-income and middle-income countries: A systematic review and meta-analysis. *Lancet Infect Dis* 2012;**12**:538–49. doi:10.1016/S1473-3099(12)70066-X

3 Cwikel JG, Lazer T, Press F, *et al.* Sexually transmissible infections among female sex workers: An international review with an emphasis on hard-to-access populations. *Sex Health* 2008;**5**:9–16. doi:10.1071/SH07024

4 Luchters S, Bosire W, Feng A, *et al.* 'A baby was an added burden': Predictors and consequences of unintended pregnancies for female sex workers in Mombasa, Kenya: A mixed-methods study. *PLoS One* 2016;**11**:1–20. doi:10.1371/journal.pone.0162871

5 Ippoliti NB, Nanda G, Wilcher R. Meeting the Reproductive Health Needs of Female Key Populations Affected by HIV in Low- and Middle-Income Countries: A Review of the Evidence. *Stud Fam Plann* 2017;**48**:121–51. doi:10.1111/sifp.12020

6 World Health Organization. WHO | Unsafe abortion: global and regional estimates of the incidence of unsafe abortion and associated mortality in 2008. 2014. doi:10.1017/CBO9781107415324.004

7 Elmore-Meegan M, Conroy RM, Agala CB. Sex Workers in Kenya, Numbers of Clients and Associated Risks: An Exploratory Survey. *Reprod Health Matters* 2004;**12**:50–7. doi:10.1016/S0968-8080(04)23125-1

8 Schwartz S, Papworth E, Thiam-Niangoin M, *et al.* An urgent need for integration of family planning services into HIV care: The high burden of unplanned pregnancy, termination of pregnancy, and limited contraception use among female sex workers in Côte d'Ivoire. *J Acquir Immune Defic Syndr* 2015;**68**:S91–8. doi:10.1097/QAI.0000000000000448

9 Bautista CT, Mejía A, Leal L, *et al.* Prevalence of lifetime abortion and methods of contraception among female sex workers in Bogota, Colombia. *Contraception* 2008;**77**:209–13. doi:10.1016/j.contraception.2007.11.013

10 Chanda MM, Ortblad KF, Mwale M, *et al.* Contraceptive use and unplanned pregnancy among female sex workers in Zambia. *Contraception* 2017;**96**:196–202. doi:10.1016/j.contraception.2017.07.003

11 Lau JTF, Mui LWH, Tsui HY, *et al.* Prevalence of induced abortion and associated factors among Chinese female sex workers in Hong Kong. *J Sex Marital Ther* 2007;**33**:19–29. doi:10.1080/00926230600998441

12 Todd CS, Alibayeva G, Sanchez JL, *et al.* Utilization of contraception and abortion and its relationship to HIV infection among female sex workers in Tashkent, Uzbekistan. *Contraception* 2006;**74**:318–23. doi:10.1016/j.contraception.2006.04.006

13 Zhang XD, Kennedy E, Temmerman M, *et al.* High rates of abortion and low levels of contraceptive use among adolescent female sex workers in Kunming, China: A cross-sectional analysis. *Eur J Contracept Reprod Heal Care* 2014;**19**:368–78. doi:10.3109/13625187.2014.927421

14 Zhang XD, Myers S, Yang HJ, *et al.* Prevalence and correlates of sexual and gender-based violence against Chinese adolescent women who are involved in commercial sex: A cross-sectional study. *BMJ Open* 2016;**6**. doi:10.1136/bmjopen-2016-013409

15 Karamouzian M, Mirzazadeh A, Shokoohi M, *et al.* Lifetime abortion of female sex workers in Iran: Findings of a national bio-behavioural survey In 2010. *PLoS One* 2016;**11**:1–12. doi:10.1371/journal.pone.0166042

16 Cleeve A, Phrasisombath K, Sychareun V, *et al.* Attitudes and experiences regarding induced abortion among female sex workers, Savannakhet Province, Laos. *Sex Reprod Healthc* 2014;**5**:137–41. doi:10.1016/j.srhc.2014.03.001

For peer review only - http://bmjopen.bmj.com/site/about/guidelines.xhtml

17 Sutherland EG, Alaii J, Tsui S, *et al.* Contraceptive needs of female sex workers in Kenya - A cross-sectional study. *Eur J Contracept Reprod Heal Care* 2011;**16**:173–82. doi:10.3109/13625187.2011.564683
18 Constitution of Kenya. Laws of kenya. *Natl Counc Law Report* 2010;:191. doi:10.1364/OE.17.019075\r186571 [pii]
19 Ushie BA, Juma K, Kimemia G, *et al.* Community perception of abortion, women who abort and abortifacients in Kisumu and Nairobi counties, Kenya. *PLoS One* 2019;**14**:1–13. doi:10.1371/journal.pone.0226120
20 Ministry of Health Republic of Kenya. Incidence and Complications of Unsafe Abortion in Kenya - Key Findings of a National Study. 2013.
21 Yi S, Tuot S, Chhoun P, *et al.* Factors associated with induced abortion among female entertainment workers: A cross-sectional study in Cambodia. *BMJ Open* 2015;**5**:1–8. doi:10.1136/bmjopen-2015-007947
22 Ampt FH, Mudogo C, Gichangi P, *et al.* WHISPER or SHOUT study: Protocol of a cluster-randomised controlled trial assessing mHealth sexual reproductive health and nutrition interventions among female sex workers in Mombasa, Kenya. *BMJ Open* 2017;**7**. doi:10.1136/bmjopen-2017-017388
23 Chemlal S, Russo G. Why do they take the risk? A systematic review of the qualitative literature on informal sector abortions in settings where abortion is legal. *BMC Womens Health* 2019;**19**:1–11. doi:10.1186/s12905-019-0751-0
24 Hall J, Barrett G, Copas A, *et al.* London Measure of Unplanned Pregnancy: guidance for its use as an outcome measure. *Patient Relat Outcome Meas* 2017;**Volume 8**:43–56. doi:10.2147/prom.s122420
25 Vyas S, Kumaranayake L. Constructing socio-economic status indices: How to use principal components analysis. *Health Policy Plan* 2006;**21**:459–68. doi:10.1093/heapol/czl029
26 Manguro GO, Gichuki C, Ampt FH, *et al.* HIV infections among female sex workers in Mombasa, Kenya: current prevalence and trends over 25 years. *Int J STD AIDS* 2020;**31**:1389–97. doi:10.1177/0956462420950571
27 Ampt FH, Lim MSC, Agius PA, *et al.* Effect of a mobile phone intervention for female sex workers on unintended pregnancy in Kenya (WHISPER or SHOUT): a cluster-randomised controlled trial. *Lancet Glob Heal* 2020;**8**:e1534–45. doi:10.1016/S2214-109X(20)30389-2
28 Ampt FH, Lim MSC, Agius PA, *et al.* Use of long-acting reversible contraception in a cluster-random sample of female sex workers in Kenya. *Int J Gynecol Obstet* 2019;**146**:184–91. doi:10.1002/ijgo.12862
29 Ampt FH, Willenberg L, Agius PA, *et al.* Incidence of unintended pregnancy among female sex workers in low-income and middle-income countries: a systematic review and meta-analysis. *BMJ Open* 2018;**8**:21779. doi:10.1136/bmjopen-2018-021779
30 Makenzius M, Faxelid E, Gemzell-danielsson K, *et al.* Contraceptive uptake in post abortion care — Secondary outcomes from a randomised controlled trial , Kisumu , Kenya. 2018;**354**:1–13.
31 Swain SN, Saggurti N, Battala M, *et al.* Experience of violence and adverse reproductive health outcomes, HIV risks among mobile female sex workers in India. *BMC Public Health* 2011;**11**:357. doi:10.1186/1471-2458-11-357
32 McDougal L, Strathdee SA, Rangel G, *et al.* Adverse Pregnancy Outcomes and Sexual Violence Among Female Sex Workers Who Inject Drugs on the United States–Mexico Border. *Violence Vict* 2013;**28**:496–512. doi:10.1891/0886-6708.11-00129
33 Campbell MK, Piaggio G, Elbourne DR, *et al.* Consort 2010 statement: Extension to cluster randomised trials. *BMJ* 2012;**345**:19–23. doi:10.1136/bmj.e5661
34 Cheuk E, Isac S, Musyoki H, *et al.* Informing HIV prevention programs for adolescent girls and young women: A modified approach to programmatic mapping and key population size estimation. *J Med Internet Res* 2019;**21**:1–11. doi:10.2196/11196

For peer review only - http://bmjopen.bmj.com/site/about/guidelines.xhtml

Figure 1: Eligibility flow diagram for the WHISPER or SHOUT study as per Consort 2010 statement: extension to cluster randomized trials.[33]

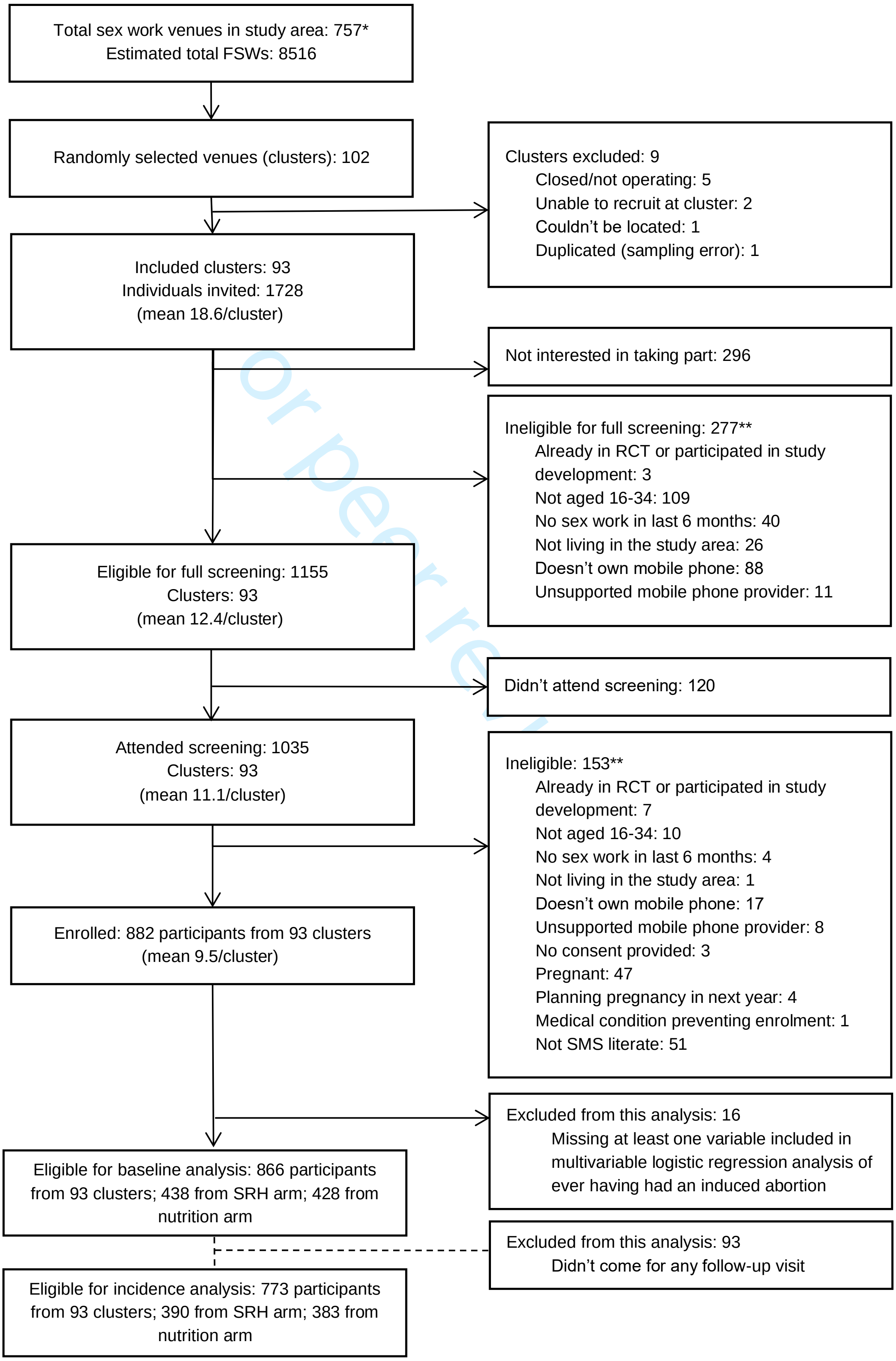

*Total hotspots and number per hotspot in study area enumerated by Cheuk et al.[34]

**One reason for ineligibility reported per participant and criteria determined in the order shown.

For peer review only - http://bmjopen.bmj.com/site/about/guidelines.xhtml

Figure 2: Overview of pregnancy outcomes during the 12-month follow-up. N=773

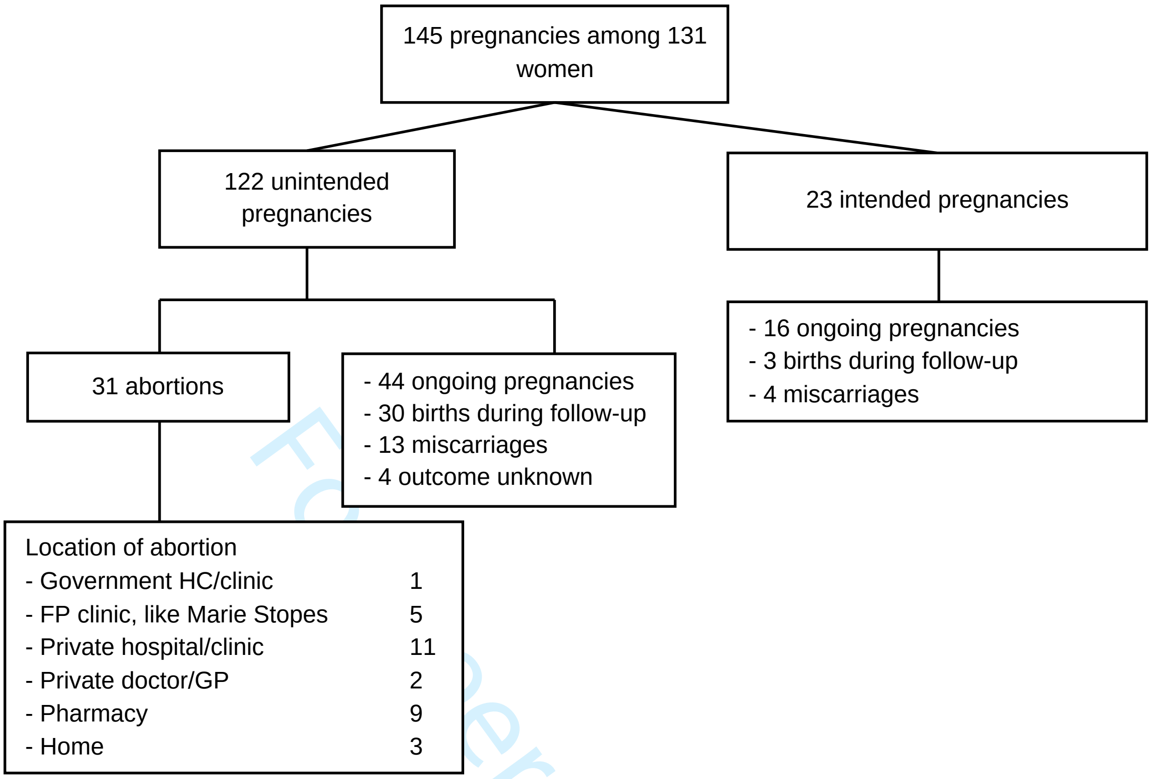

For peer review only

STROBE Statement—Checklist of items that should be included in reports of ***cohort studies***

Protocol paper [1]:

1 Ampt FH, Mudogo C, Gichangi P, *et al.* WHISPER or SHOUT study: Protocol of a cluster-randomised controlled trial assessing mHealth sexual reproductive health and nutrition interventions among female sex workers in Mombasa, Kenya. *BMJ Open* 2017;**7**. doi:10.1136/bmjopen-2017-017388

	Item No	Recommendation	Page No
**Title and abstract**	1	(*a*) Indicate the study's design with a commonly used term in the title or the abstract	**1**
		(*b*) Provide in the abstract an informative and balanced summary of what was done and what was found	**2**
**Introduction**			
Background/rationale	2	Explain the scientific background and rationale for the investigation being reported	**5**
Objectives	3	State specific objectives, including any prespecified hypotheses	**5**
**Methods**			
Study design	4	Present key elements of study design early in the paper	**6**
Setting	5	Describe the setting, locations, and relevant dates, including periods of recruitment, exposure, follow-up, and data collection	**6 + Ref [1] to protocol paper**
Participants	6	(*a*) Give the eligibility criteria, and the sources and methods of selection of participants. Describe methods of follow-up	**6 + Ref [1] to protocol paper**
		(*b*) For matched studies, give matching criteria and number of exposed and unexposed	
Variables	7	Clearly define all outcomes, exposures, predictors, potential confounders, and effect modifiers. Give diagnostic criteria, if applicable	**6, 7**
Data sources/ measurement	8*	For each variable of interest, give sources of data and details of methods of assessment (measurement). Describe comparability of assessment methods if there is more than one group	**6, 7**
Bias	9	Describe any efforts to address potential sources of bias	**Ref [1] to protocol paper**
Study size	10	Explain how the study size was arrived at	**Ref [1] to protocol paper**
Quantitative variables	11	Explain how quantitative variables were handled in the analyses. If applicable, describe which groupings were chosen and why	**7, 8**
Statistical methods	12	(*a*) Describe all statistical methods, including those used to control for confounding	**7, 8**
		(*b*) Describe any methods used to examine subgroups and interactions	
		(*c*) Explain how missing data were addressed	
		(*d*) If applicable, explain how loss to follow-up was addressed	
		(*e*) Describe any sensitivity analyses	

For peer review only

For peer review only - http://bmjopen.bmj.com/site/about/guidelines.xhtml

Results			
Participants	13*	(a) Report numbers of individuals at each stage of study—eg numbers potentially eligible, examined for eligibility, confirmed eligible, included in the study, completing follow-up, and analysed<br>(b) Give reasons for non-participation at each stage<br>(c) Consider use of a flow diagram	**6 + Figure 1**
Descriptive data	14*	(a) Give characteristics of study participants (eg demographic, clinical, social) and information on exposures and potential confounders<br>(b) Indicate number of participants with missing data for each variable of interest<br>(c) Summarise follow-up time (eg, average and total amount)	**9, 10, 11 + Table 1**
Outcome data	15*	Report numbers of outcome events or summary measures over time	**9, 11**

For peer review only

Main results	16	(*a*) Give unadjusted estimates and, if applicable, confounder-adjusted estimates and their precision (eg, 95% confidence interval). Make clear which confounders were adjusted for and why they were included<br>(*b*) Report category boundaries when continuous variables were categorized<br>(*c*) If relevant, consider translating estimates of relative risk into absolute risk for a meaningful time period	**9-12 + Tables 2 and 3**
Other analyses	17	Report other analyses done—eg analyses of subgroups and interactions, and sensitivity analyses	**12, Table 3**
**Discussion**			
Key results	18	Summarise key results with reference to study objectives	**13**
Limitations	19	Discuss limitations of the study, taking into account sources of potential bias or imprecision. Discuss both direction and magnitude of any potential bias	**14, 15**
Interpretation	20	Give a cautious overall interpretation of results considering objectives, limitations, multiplicity of analyses, results from similar studies, and other relevant evidence	**13-15**
Generalisability	21	Discuss the generalisability (external validity) of the study results	**15**
**Other information**			
Funding	22	Give the source of funding and the role of the funders for the present study and, if applicable, for the original study on which the present article is based	**2-3, 16**

*Give information separately for exposed and unexposed groups.

Note: An Explanation and Elaboration article discusses each checklist item and gives methodological background and published examples of transparent reporting. The STROBE checklist is best used in conjunction with this article (freely available on the Web sites of PLoS Medicine at http://www.plosmedicine.org/, Annals of Internal Medicine at http://www.annals.org/, and Epidemiology at http://www.epidem.com/). Information on the STROBE Initiative is available at http://www.strobe-statement.org.

For peer review only

For peer review only - http://bmjopen.bmj.com/site/about/guidelines.xhtml
